# Supplementary material for: RAC1B modulates intestinal tumourigenesis via modulation of WNT and EGFR signalling pathways
Source: Nat Commun. 2021 Apr 20;12:2335. doi: 10.1038/s41467-021-22531-3 (PMC8058071; doi:10.1038/s41467-021-22531-3)
Supplement: Supplementary file 1 — Supplementary Information [file 41467_2021_22531_MOESM1_ESM.docx]

**Supplementary Table 1**

**qRT-PCR primers used in this study:**

|  | **Forward (5'-3')** | **Reverse (5'-3')** |
| --- | --- | --- |
| ***Axin2*** | TGACTCTCCTTCCAGATCCCA | TGCCCACACTAGGCTGACA |
| ***Ccnd1*** | GCGTACCCTGACACCAATCTC | CTCCTCTTCGCACTTCTGCTC |
| ***Ephb2*** | GCGGCTACGACGAGAACAT | GGCTAAGTCAAAATCAGCCTCA |
| ***Etv4*** | CGGAGGATGAAAGGCGGATAC | TCTTGGAAGTGACTGAGGTCC |
| ***Etv5*** | TCAGTCTGATAACTTGGTGCTTC | GGCTTCCTATCGTAGGCACAA |
| ***Fzd3*** | ATGGCTGTGAGCTGGATTGTC | GGCACATCCTCAAGGTTATAGGT |
| ***hRac1b*** | CAGTAAGGGAGCTGCAGTGG | GCGAAGAGTTTGTCCTCAACC |
| ***Krt20*** | AGTTTTCACCGAAGTCTGAGTTC | GTAGCTCATTACGGCTTTGGAG |
| ***Lef1*** | TGTTTATCCCATCACGGGTGG | CATGGAAGTGTCGCCTGACAG |
| ***Lgr5*** | GAGTCAACCCAAGCCTTAGTATCC | CATGGGACAAATGCAACTGAAG |
| ***Myc*** | ATGCCCCTCAACGTGAACTTC | CGCAACATAGGATGGAGAGCA |
| ***Rac1*** | GAGACGGAGCTGTTGGTAAAA | ATAGGCCCAGATTCACTGGTT |
| ***Rac1b*** | TGTGGTAAAGATAGACCCTCC | CCCACGAGGATGATAGGAGT |
| ***Tiam1*** | GAAGCACACTTCACGCTCC | CTCCAGGCCATTTTCAGCCA |
| ***Tnfrsf19*** | TTCTGTGGGGGACACGATG | AGAAAATTCAGCGCAGATGGAA |
| ***Znrf3*** | GGCGACTATACCACCCACAC | CTTCACCACTCCTACCCAGC |
| ***β-actin*** | GTGACGTTGACATCCGTAAAGA | GCCGGACTCATCGTACTCC |

**Supplementary Figures**

**
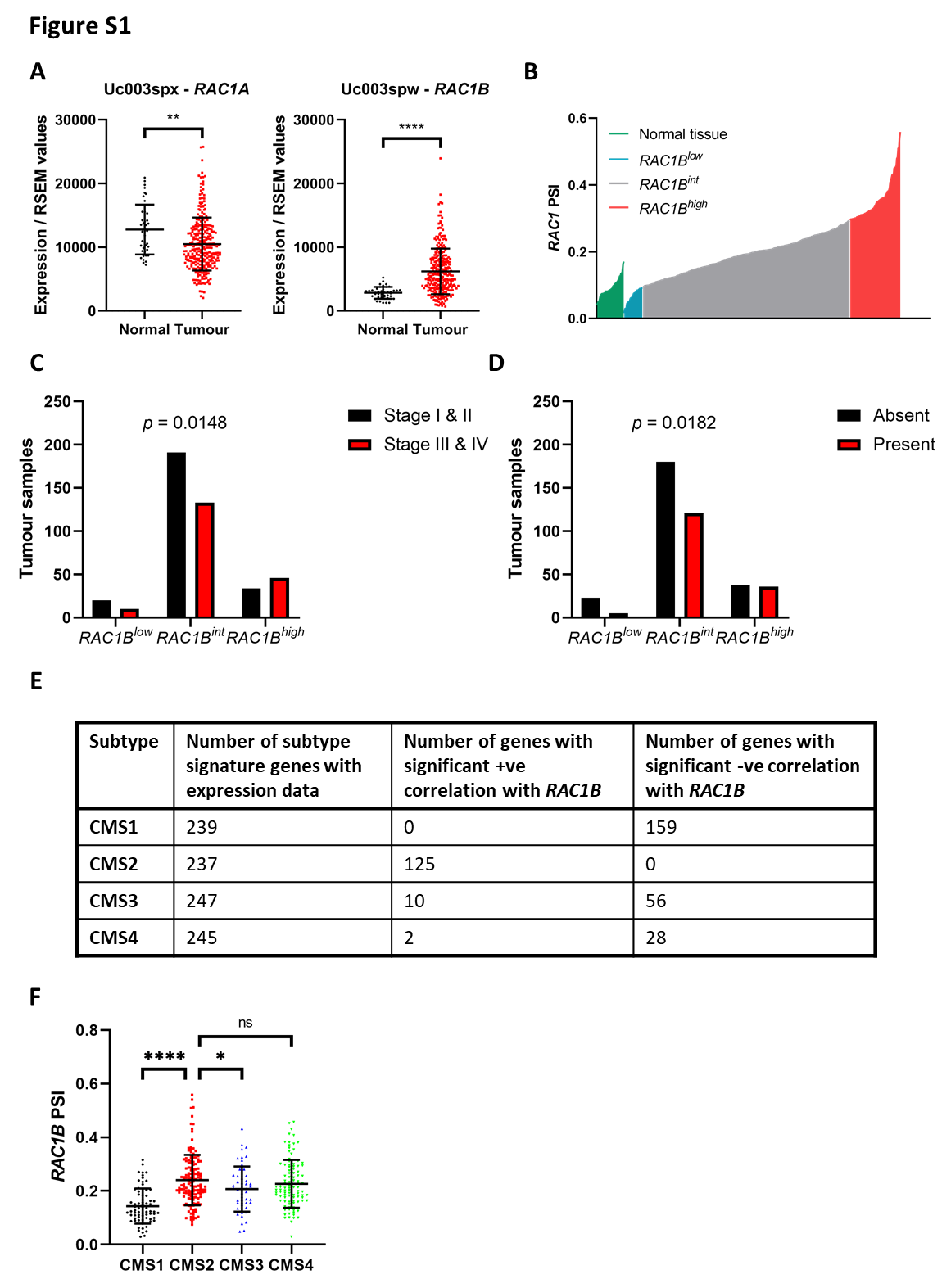
**

**
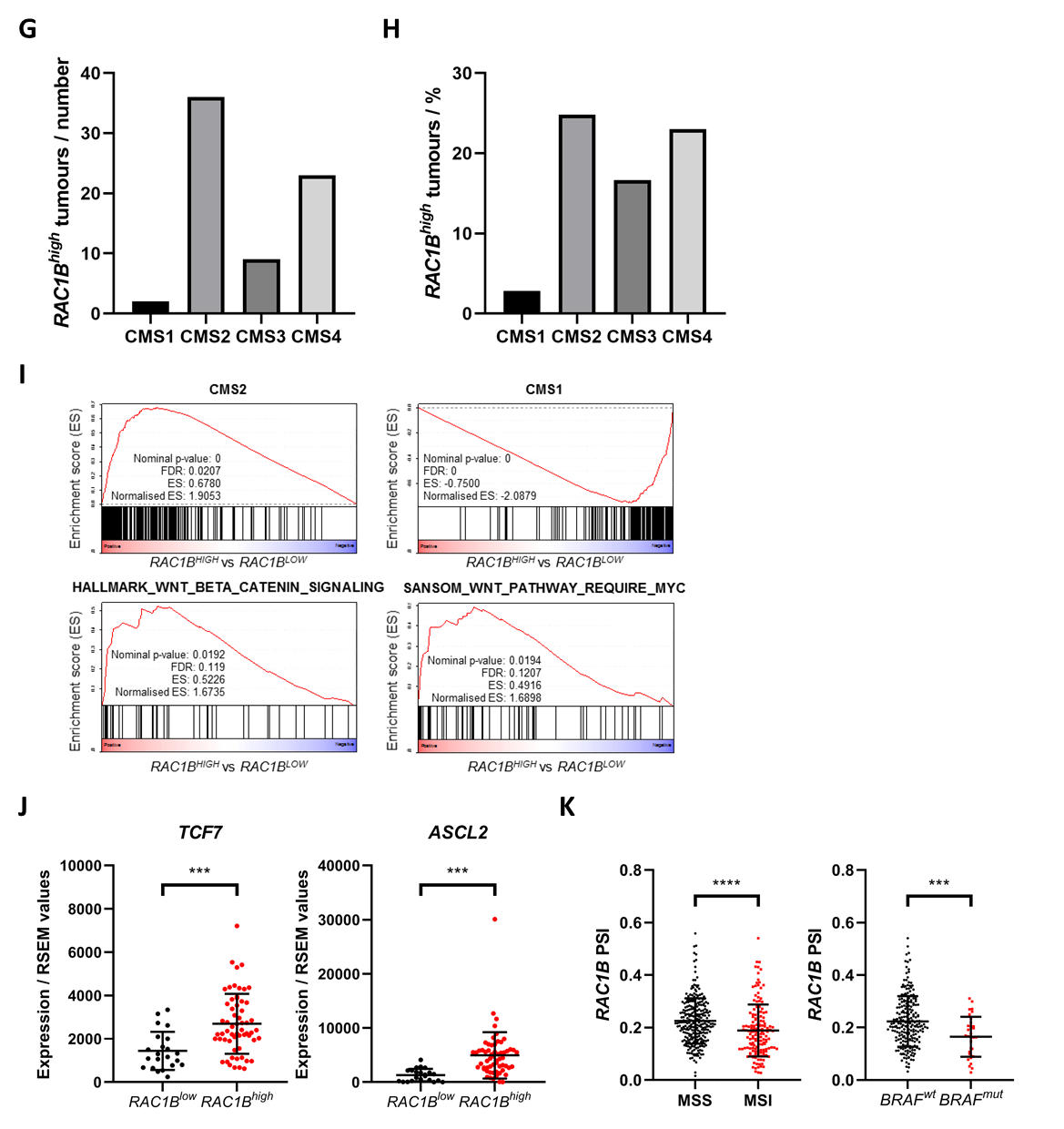
**

**Figure S1. *RAC1B* overexpression in human colorectal cancer.** (A) Individual value plots of transcripts Uc003spx - *RAC1A* (left panel) and Uc003spw - *RAC1B* (right panel) isoform expression comparing normal and colorectal tumour tissue (TCGA COAD dataset). *RAC1;* data are presented as mean values +/- SD; ***P* = 0.001; two tailed t test, n = 41 vs 288 tumour samples). *RAC1B;* data are presented as mean values +/- SD; *****P* = 6.18 x 10^-9^; two tailed t test, n = 41 vs 288 tumour samples). (B) Distribution of *RAC1B* PSI values across TCGA dataset. Expression values for normal tissue (green), *RAC1B^low^* (blue), *RAC1B^int^* (grey) and *RAC1B^high^* (red) are highlighted. (C) Bar graph showing the number of *RAC1B^low^, RAC1B^int^* and *RAC1B^high^* tumours in patients with early or late stage disease (Chi-square test shown). (D) Bar graph showing the number of *RAC1B^low^, RAC1B^int^* and *RAC1B^high^* tumours in patients where lymphovascular invasion was present or absent (Chi-square test shown). (E) Table outlining the number of significant correlations between *RAC1B* expression and CMS signature genes for each subtype. (F) Individual value plot of *RAC1B* PSI values in different CMS subtypes (data are presented as mean values +/- SD; *****P* = 1.13 x 10^-13^, **P* = 0.0159; one way anova with FDR multiple correction. (G) Number of *RAC1B^high^* tumours in each CMS (H) Percentage of *RAC1B^high^* tumours in each CMS. (I) GSEA plots of various CMS and Wnt target gene sets comparing *RAC1B^high^* versus *RAC1B^low^* tumours from TCGA dataset (p-value, FDR, enrichment score and normalised enrichment score shown). (J) Individual value plots of WNT target gene expression in tumours expressing low versus high *RAC1B* levels. *TCF7;* data are presented as mean values +/- SD; ****P* = 0.00017; two tailed t test, n = 22 vs 60 tumour samples). *ASCL2;* data are presented as mean values +/- SD; ****P* = 0.00017; two tailed t test, n = 22 vs 60 tumour samples). (K) Individual value plots of *RAC1B* expression in microsatellite stable (MSS) versus MSI tumours (left panel) and in tumours wildtype or mutated for *BRAF* (right panel). MSI; data are presented as mean values +/- SD; *****P* = 9.23 x 10^-5^; two tailed t test, n = 268 vs 151 tumour samples). *BRAF;* data are presented as mean values +/- SD; ****P* = 0.0009; two tailed t test, n = 223 vs 33 tumour samples). Source data are provided as a Source Data file.

**
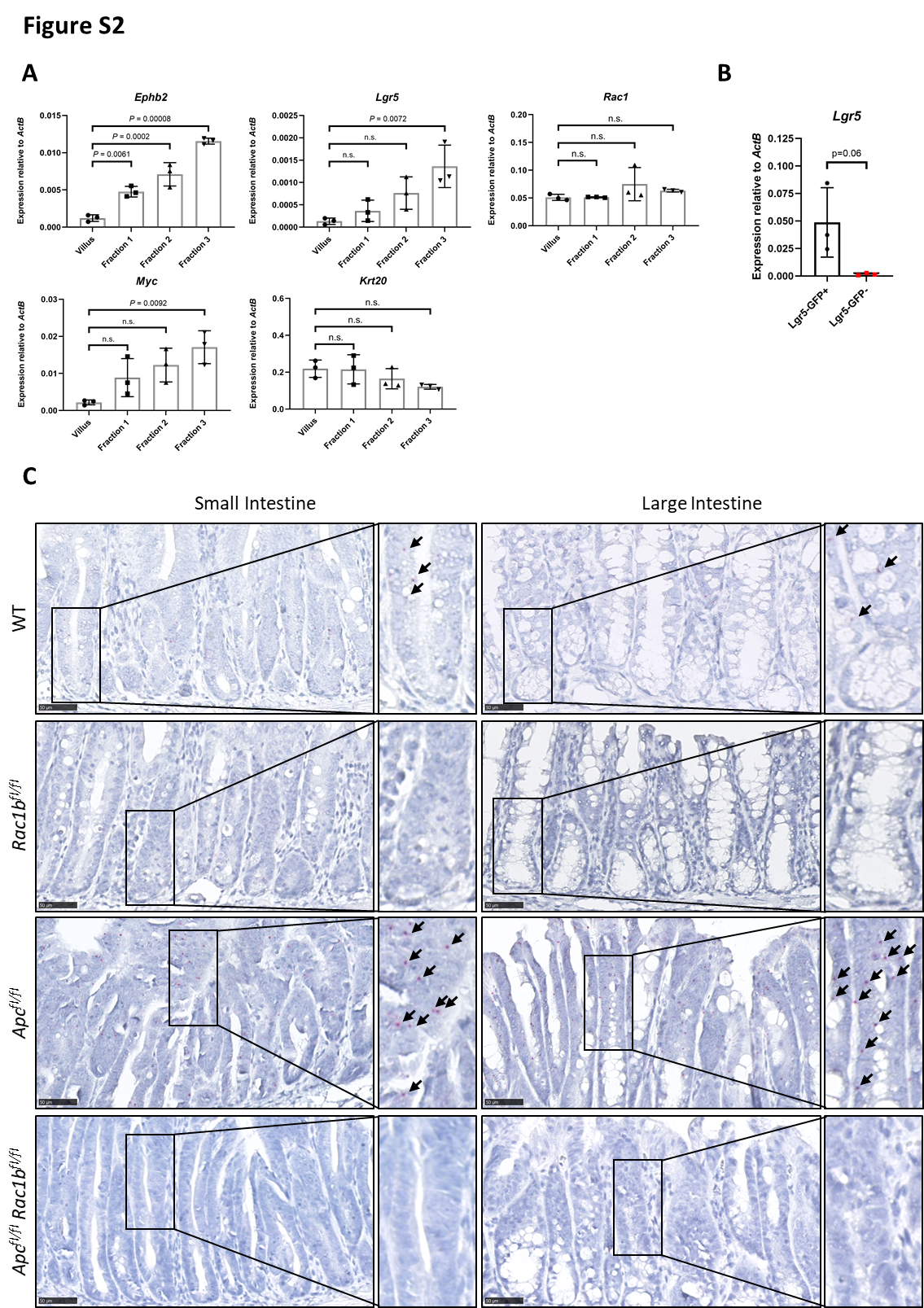

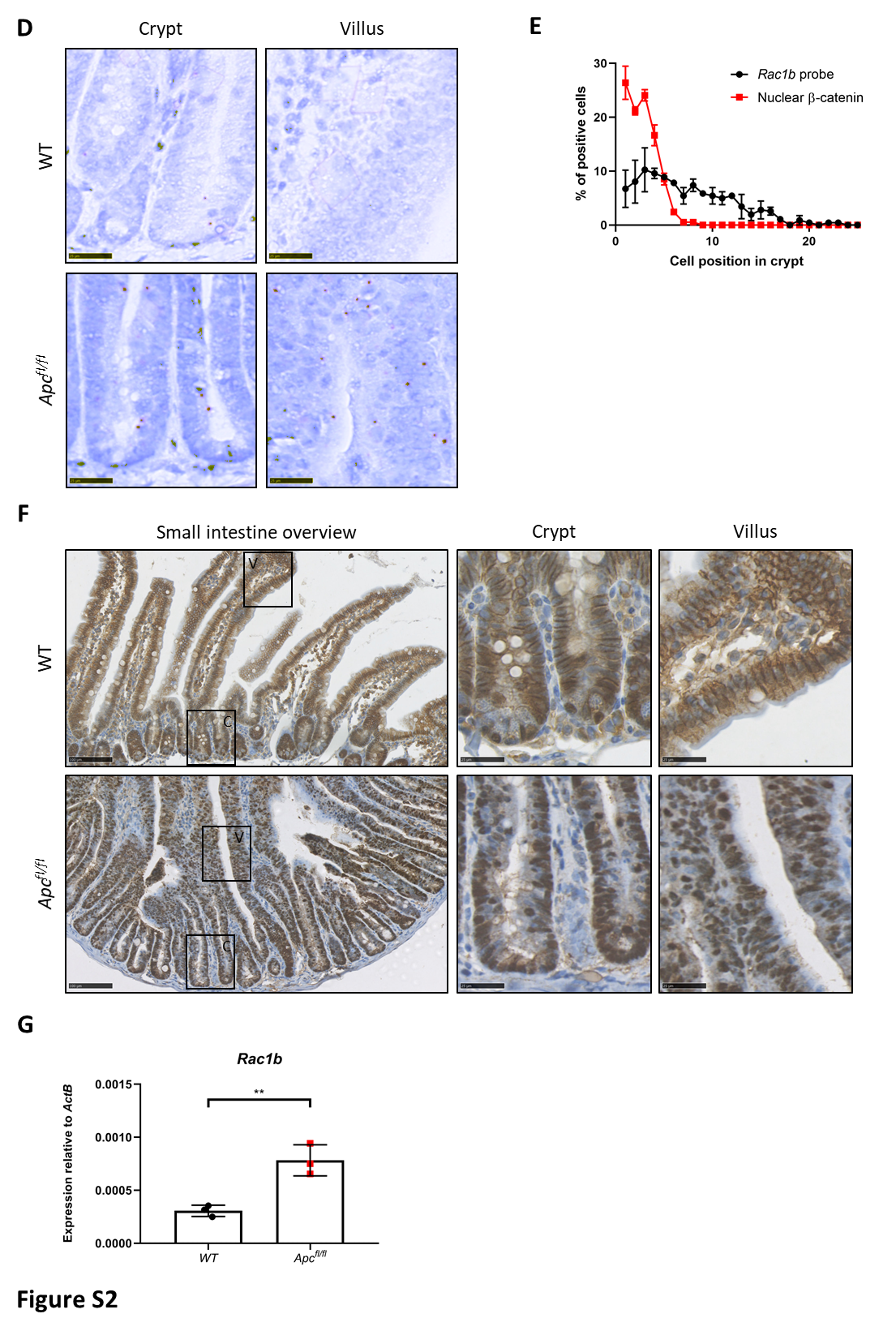
**

**Figure S2. *Rac1b* is expressed in intestinal crypt cells and its expression increases following *Apc* deletion.** (A) qRT-PCR of stem, progenitor and differentiation markers and *Rac1* from villus and crypt fractions data are presented as mean values +/- SD; *P<0.05, **P < 0.01, ****P < 0.0001; one way anova with Tukey multiple correction, n = fractions from 3 mice). (B) qRT-PCR of *Lgr5* from Lgr5+ and Lgr5- cells (data are presented as mean values +/- SD, P=0.06; two tailed t test, n = 3 biologically independent mice). (C) *Rac1b* basescope analysis of *WT, Rac1b^fl/fl^, Apc^fl/fl^* and *Apc^fl/fl^* Rac1b*^fl/fl^* small and large intestinal tissue. Magnified areas are shown in right panels. Black arrows indicate positive probe detection (pink dots). Scale bars are 50µm. Basescope analysis was carried out by staining of intestinal samples derived from 3 vs 3 vs 3 vs 3 biologically independent mice. (D) False colour images of images presented in Fig 2D to enhance visibility of Basescope dots. These are representative images of Fig 2D, false colour processing was carried out only on the images shown. (E) Positional scoring of *Rac1b* probe positivity and β-catenin nuclear localisation in *WT* small intestinal tissue. Note the extend crypt localisation of *Rac1b* probe compared to the crypt base position of β-catenin nuclear localisation data are presented as mean values +/- SEM, n = 3 mice). (F) β-catenin IHC in *WT* and *Apc^fl/fl^* small intestine. Scale bars are 100µm (overview) and 25µm (zoom). IHC analysis was carried out on intestinal samples derived from 3 vs 3 biologically independent mice. (G) qRT-PCR of *Rac1b* from *WT* versus *Apc^fl/fl^* intestinal tissue (data are presented as mean values +/- SD; ***P* = 0.0062; two tailed t test, n = 3 vs 3 biologically independent mice). Source data are provided as a Source Data file.

**
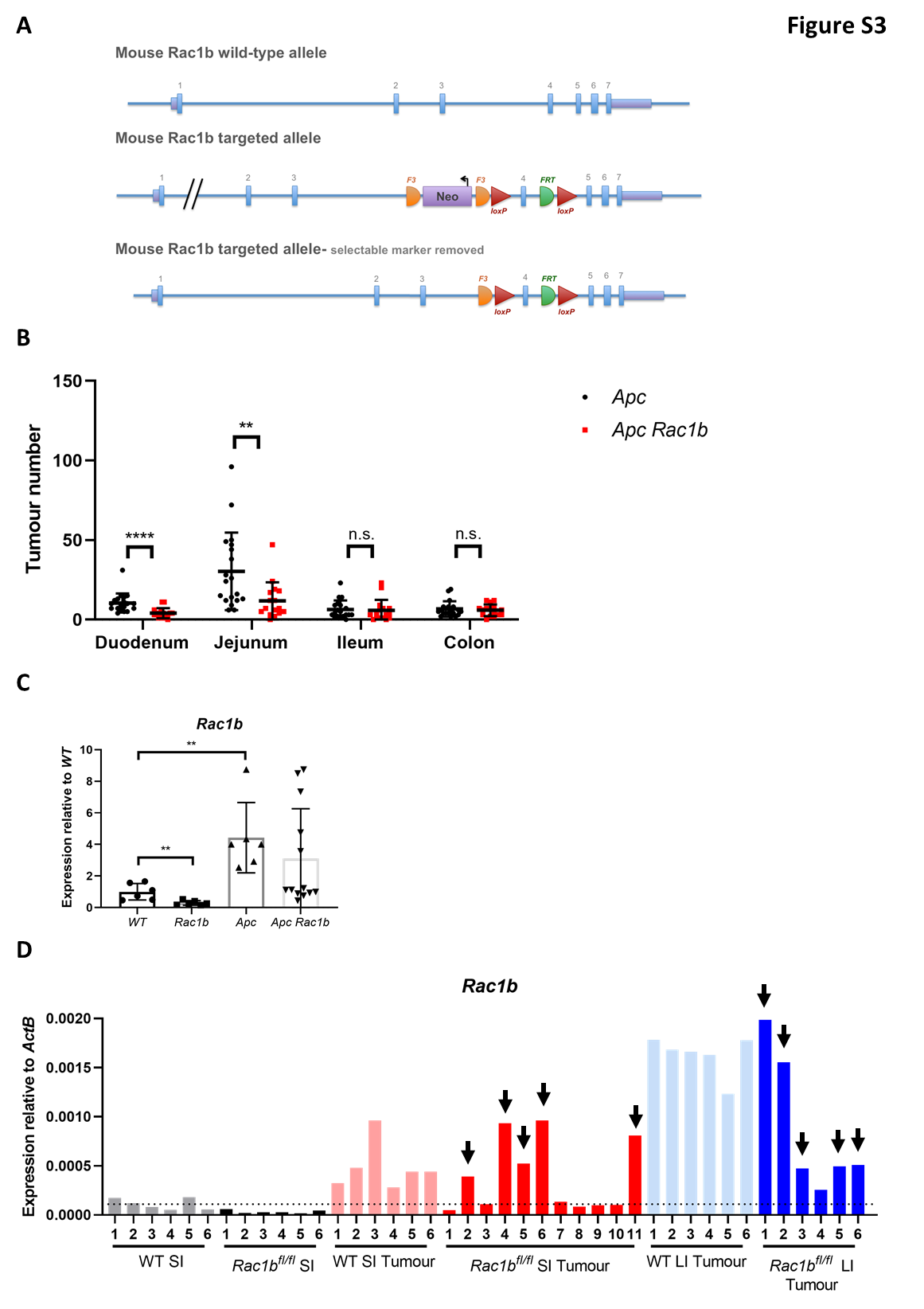

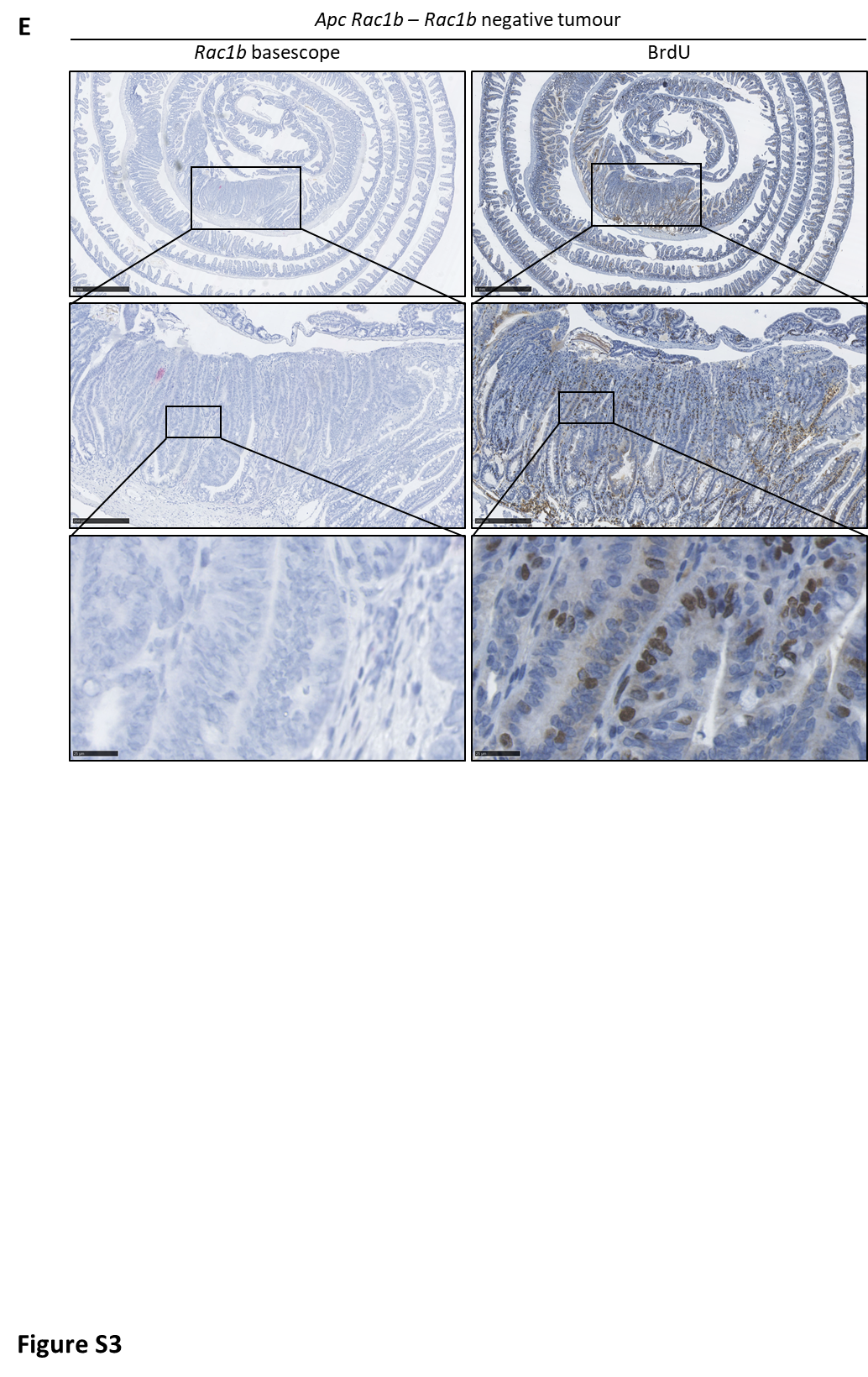

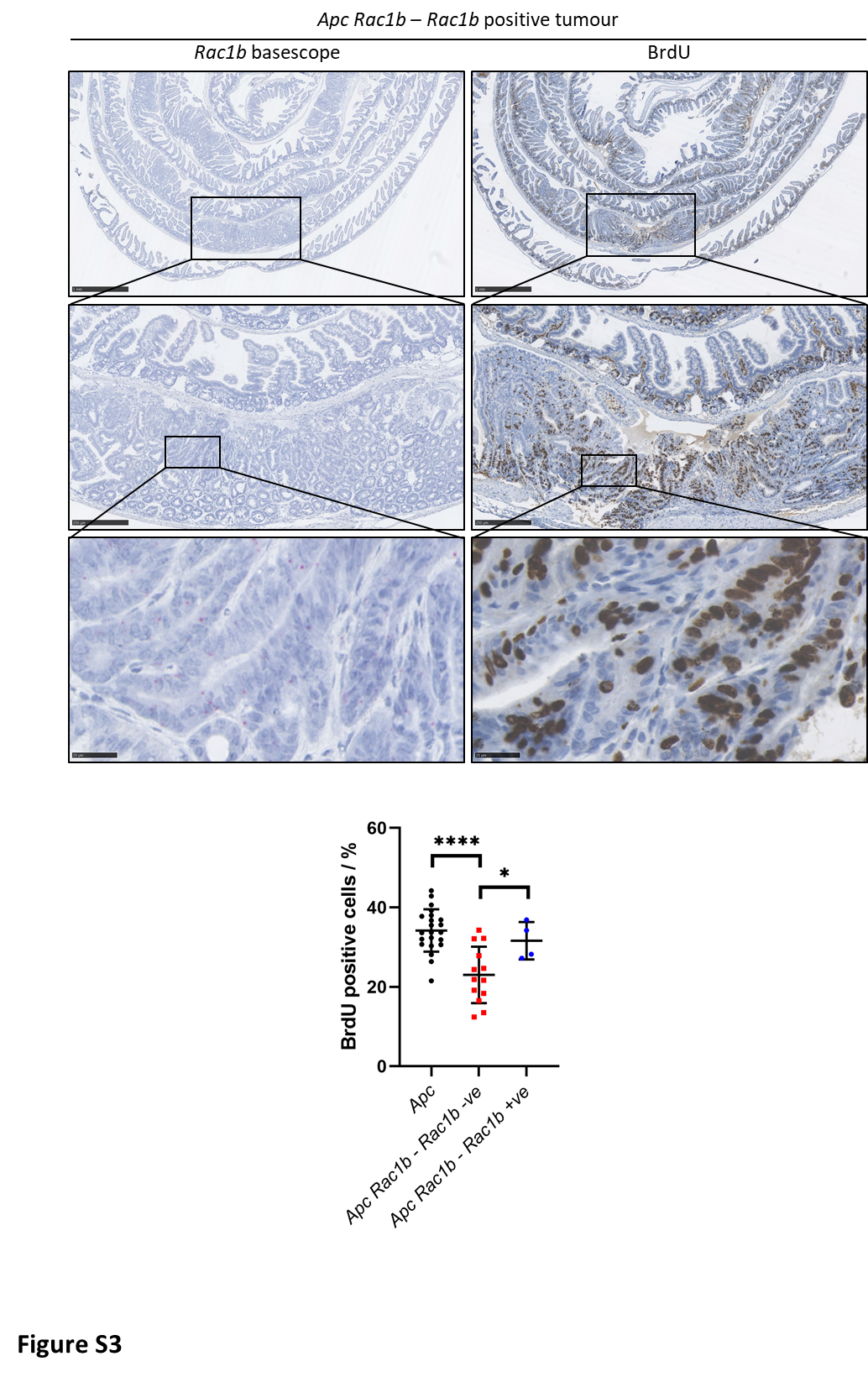
h
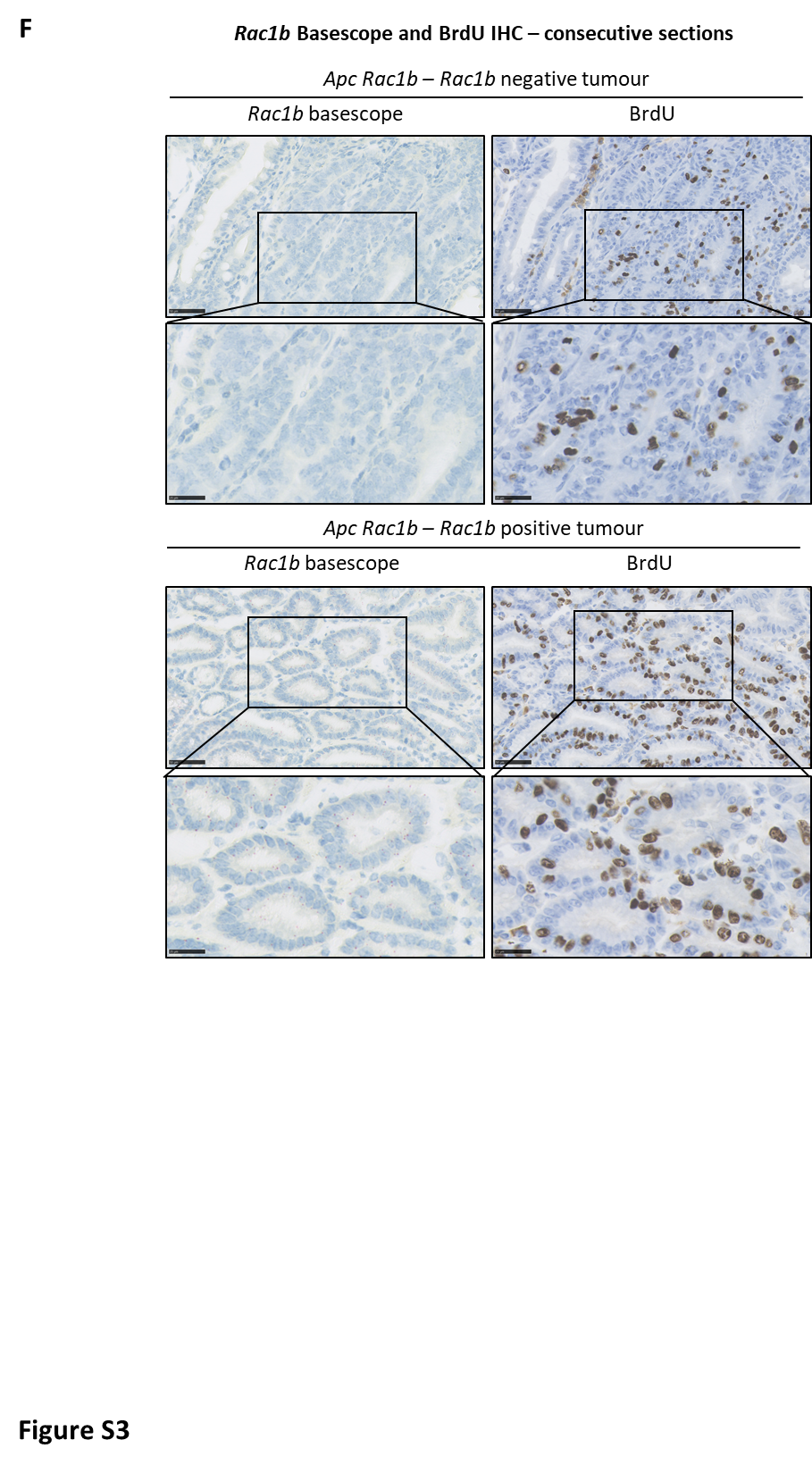

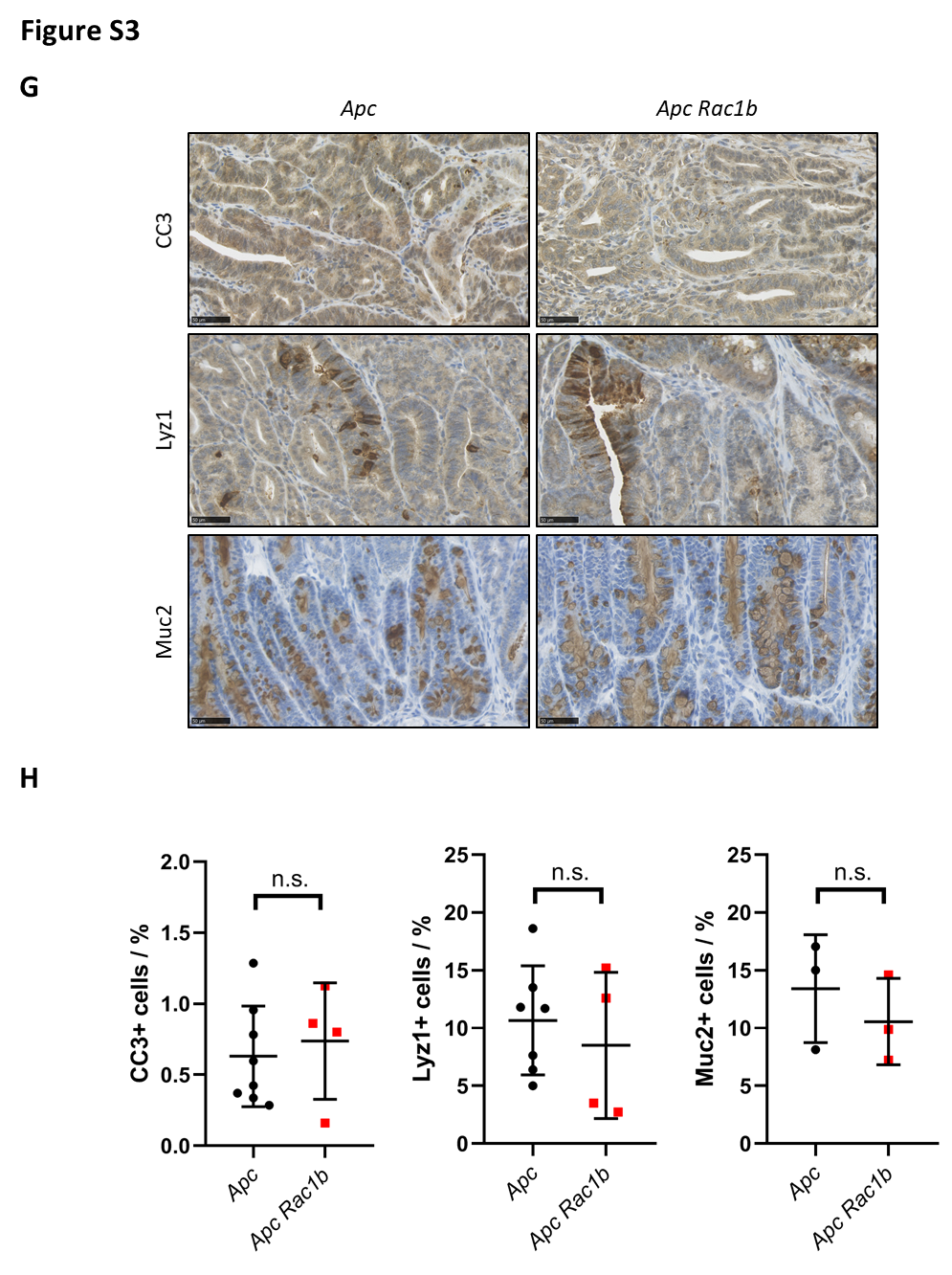

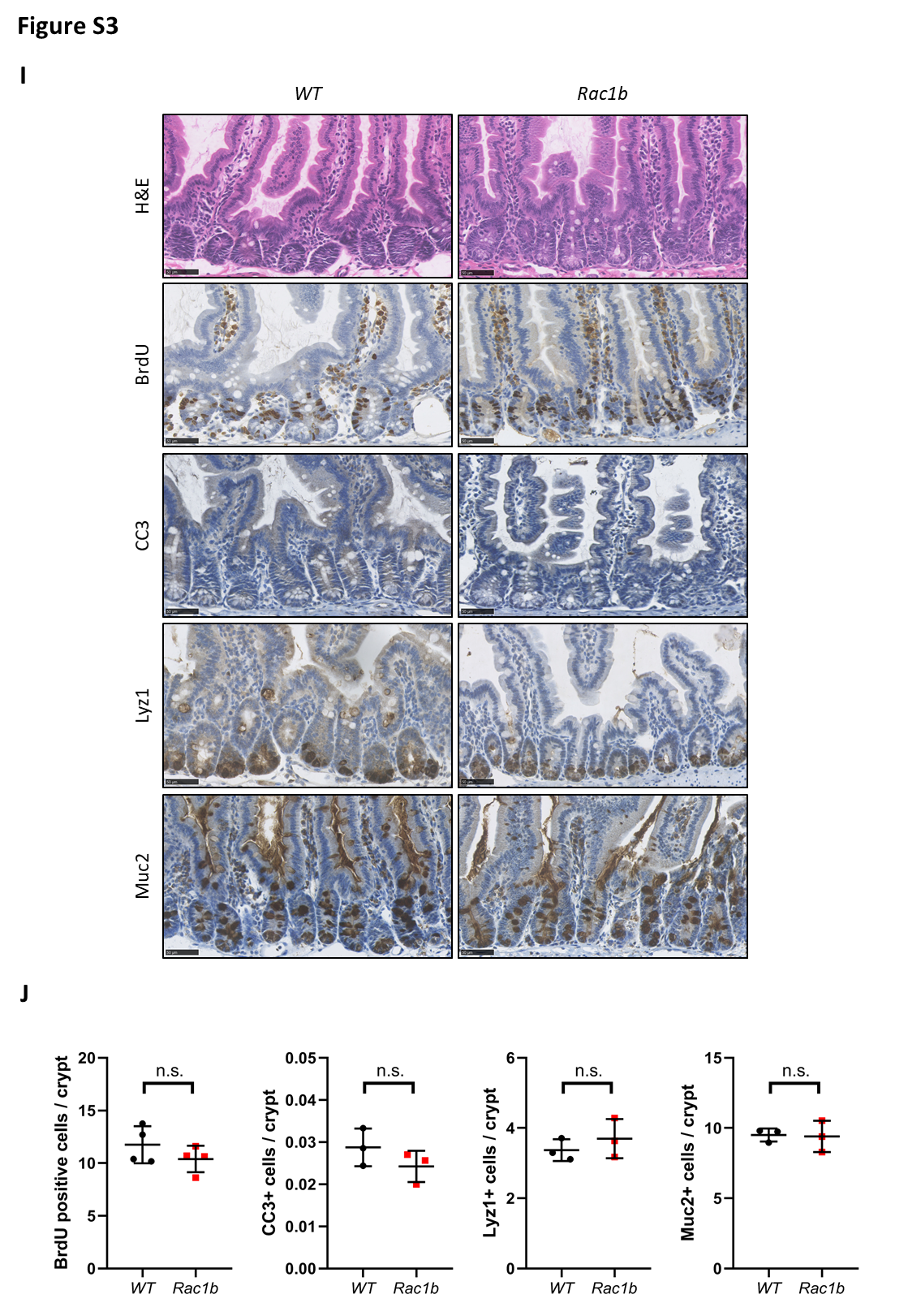

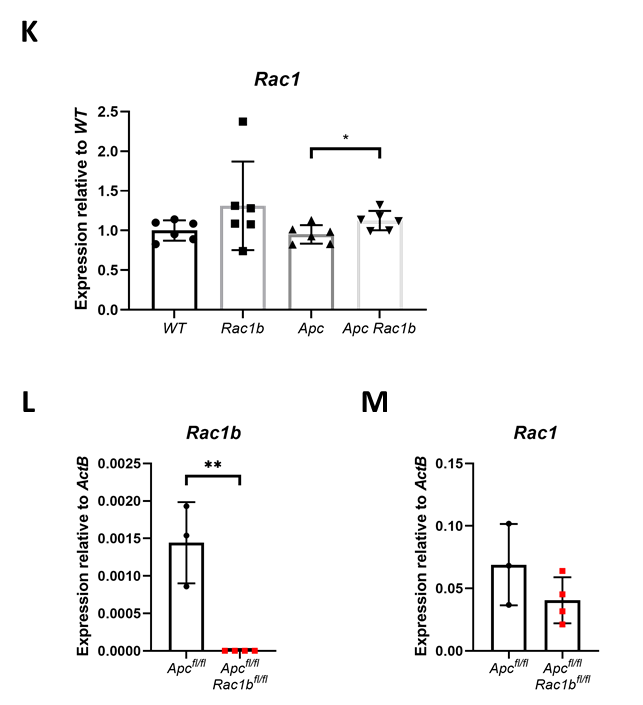
**

**Figure S3. Deletion of *Rac1b* suppresses intestinal tumourigenesis.** (A) Schematic outlining the targeting strategy utilised to generate the *Rac1b* floxed allele. (B) Quantification of tumour number in different regions of the intestine of *Apc* versus *Apc Rac1b* mice (data are presented as mean values +/- SD; ***P* = 0.0074, *****P* = 0.00002; two tailed Mann-Whitney test, n = 19 vs 16 biologically independent mice). (C) qRT-PCR analysis of *Rac1b* expression in matched normal SI and SI tumour tissue from *Apc* and *Apc Rac1b* mice (normal SI from *Apc* mice designated *WT* and normal tissue from *Apc Rac1b* mice designated *Rac1b*). data are presented as mean values +/- SD; ***P* = 0.0093 (*WT* vs *Rac1b*), ***P* = 0.0044 (*WT* vs *Apc*); two tailed t test, n = 6 vs 6 vs 6 vs 13 tumours from biologically independent mice. (D) Bar plot showing expression level of individual samples from indicated genotypes. Arrows indicate tumours from *Apc Rac1b* mice that express *Rac1b*. Dashed line indicates average *Rac1b* expression level in *WT* SI samples. Each bar is an individual tumour sample. (E) *Rac1b* basescope and BrdU IHC analysis of tumours from *Apc Rac1b* mice. A *Rac1b* negative (top panels) and *Rac1b* positive (bottom panels) tumour is shown. Magnified areas are shown below. Scale bars are 250µm (overview) and 25µm (inset). Note the Basescope positivity (pink dots) in the positive tumour. Individual value plot of quantification of BrdU incorporation in *Rac1b* positive and negative tumours (data are presented as mean values +/- SD; *****P* = 1.39 x 10^-5^, **P* = 0.0409; one way ANOVA, n = 22 vs 13 vs 4 tumours from biologically independent mice). (F) Sequential images of tumours stained for *Rac1b* Basescope and BrdU IHC. These are representative images from analyses carried out on 3 vs 3 biologically independent mice. (G) Histological analysis of tumours from *Apc* and *Apc Rac1b* mice. Images of cleaved caspase 3 (CC3), Lyz1 and Muc2 stained intestines are shown (Scale bars = 50µm). *Apc Rac1b* tumours analysed in this experiment were confirmed to be *Racb1* negative by qRT-PCR. (H) Quantification of CC3, Lyz1 and Muc2 stained *Apc* and *Apc Rac1b* tumours respectively (data are presented as mean values +/- SD; P=not significant; two tailed t test; n = 8 vs 4 biologically independent mice (CC3), n = 7 v 4 biologically independent mice (Lyz1) and n = 3 v 3 biologically independent mice (Muc2). (I) Histological analysis of *WT* and *Rac1b* intestine. Images of H&E, BrdU, cleaved caspase 3 (CC3), Lyz1 and Muc2 stained intestines are shown (Scale bar = 50µm). (J) Quantification of BrdU incorporation, CC3, Lyz1 and Muc2 stained *WT* and *Rac1b* intestines respectively (data are presented as mean values +/- SD; P=not significant; two tailed t test; n = 4 v 4 biologically independent mice (BrdU), n = 3 v 3 biologically independent mice (CC3), n = 3 v 3 biologically independent mice (Lyz1) and n = 3 v 3 biologically independent mice (Muc2)). (K) qRT-PCR analysis of *Rac1* expression in matched normal SI and SI tumour tissue from *Apc* and *Apc Rac1b* mice. Data are presented as mean values +/- SD; **P* = 0.0311; two tailed t test, n = 6 vs 6 vs 6 vs 6 biologically independent mice. (L) qRT-PCR analysis of *Rac1b* expression in *Apc^fl/fl^* and *Apc^fl/fl^ Rac1b^fl/fl^* organoids. Data are presented as mean values +/- SD; ***P* = 0.0027; two tailed t test, n = 3 vs 4 biologically independent mice. (M) qRT-PCR analysis of *Rac1* expression in *Apc^fl/fl^* and *Apc^fl/fl^ Rac1b^fl/fl^* organoids. Data are presented as mean values +/- SD; n = 3 vs 4 biologically independent mice. Source data are provided as a Source Data file.

**
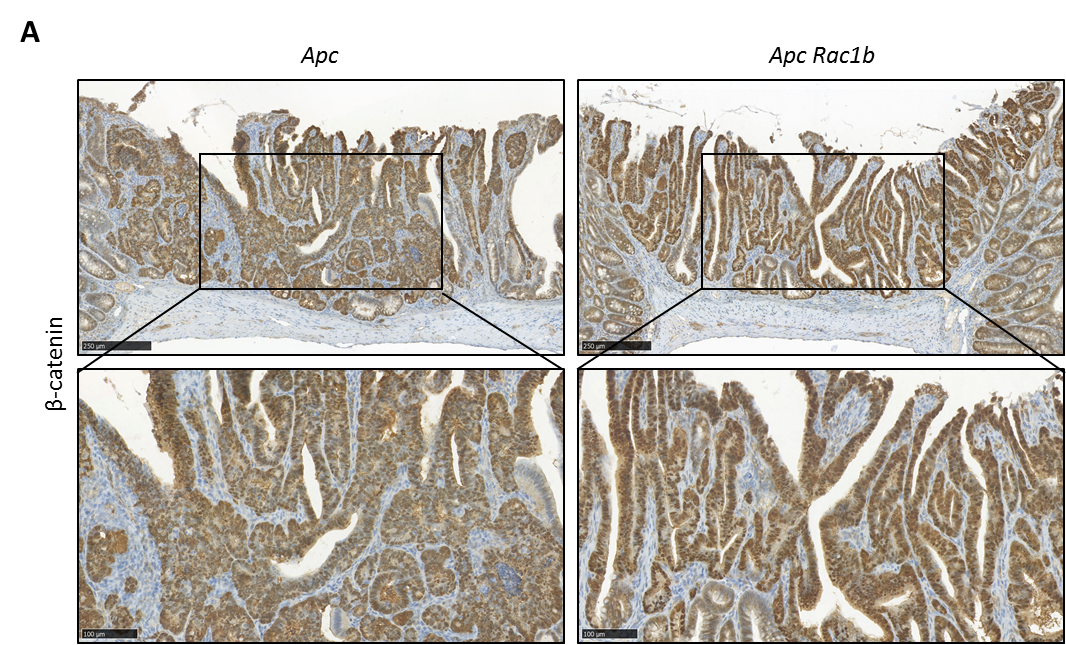
**

**Figure S4. Histological analysis of *Rac1b* knockout intestinal tumours.** (A) β-catenin IHC of *Apc* and *Apc Rac1b* SI tumour. Magnified areas are shown in panels demonstrating nuclear staining. Scale bars are 250µm (top panels) and 100µm (magnified). *Apc Rac1b* tumours analysed in this experiment were confirmed to be *Racb1* negative by qRT-PCR. ). IHC analysis was carried out on intestinal samples derived from 3 vs 3 biologically independent mice.

**
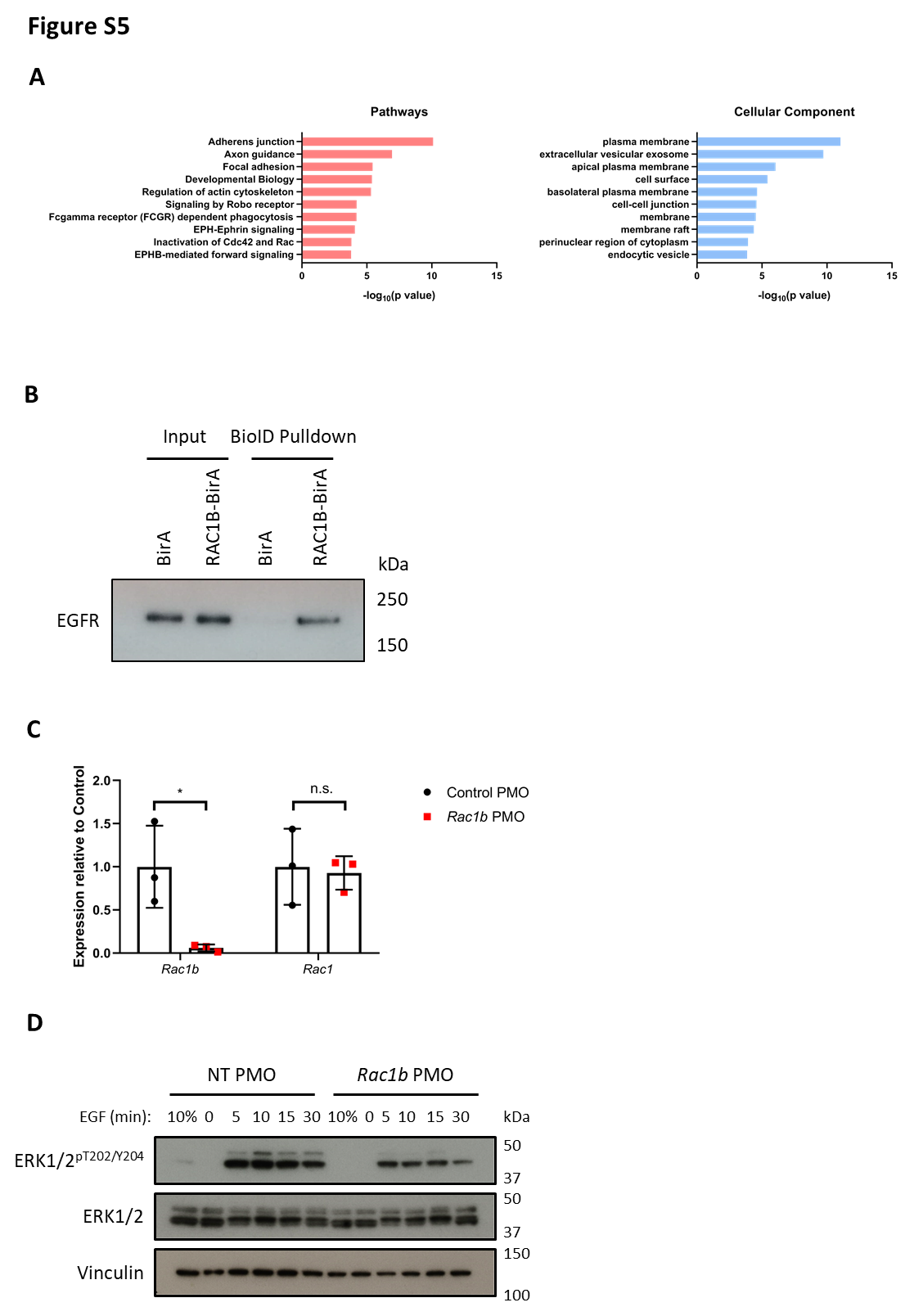

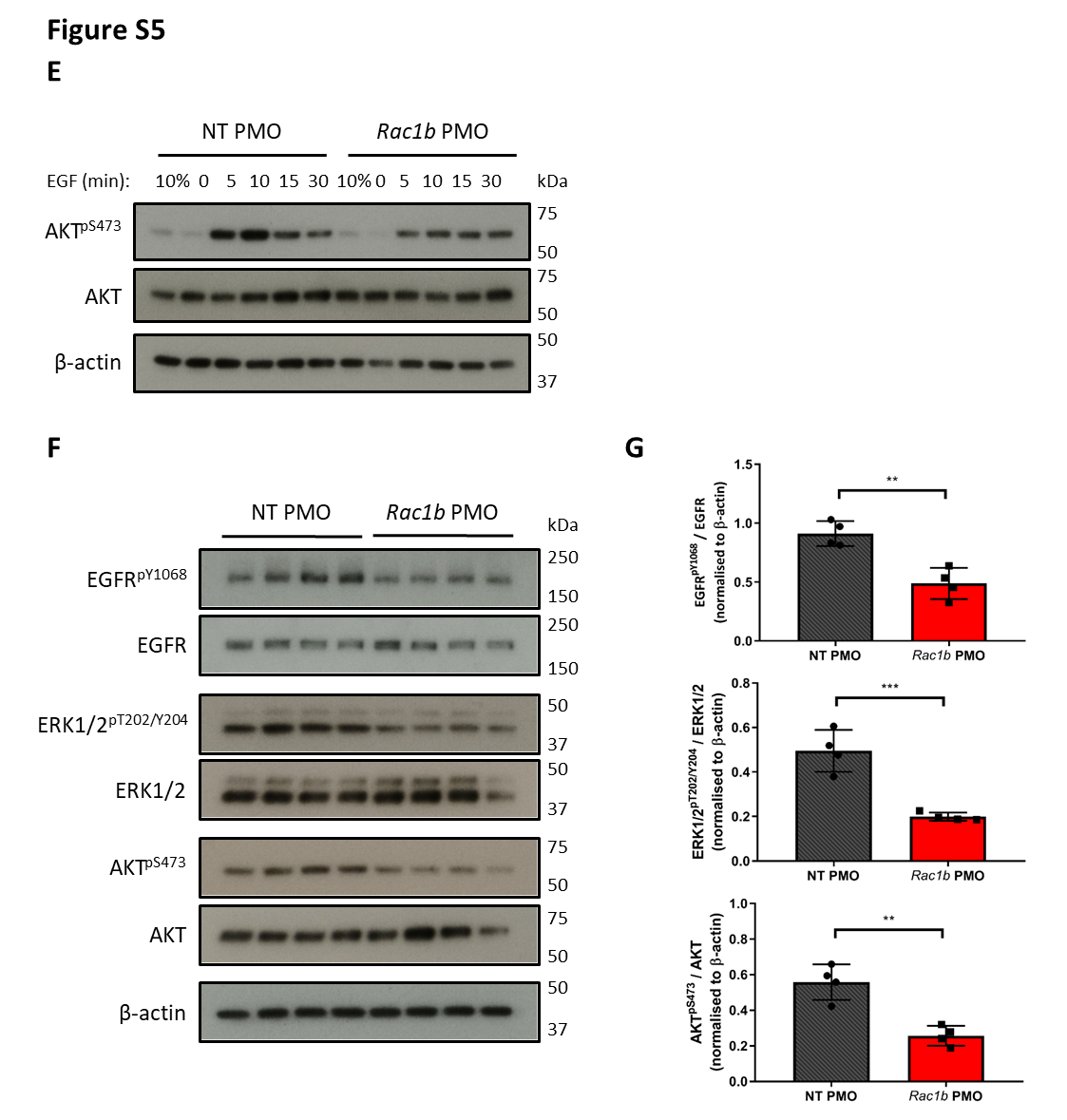
**

**
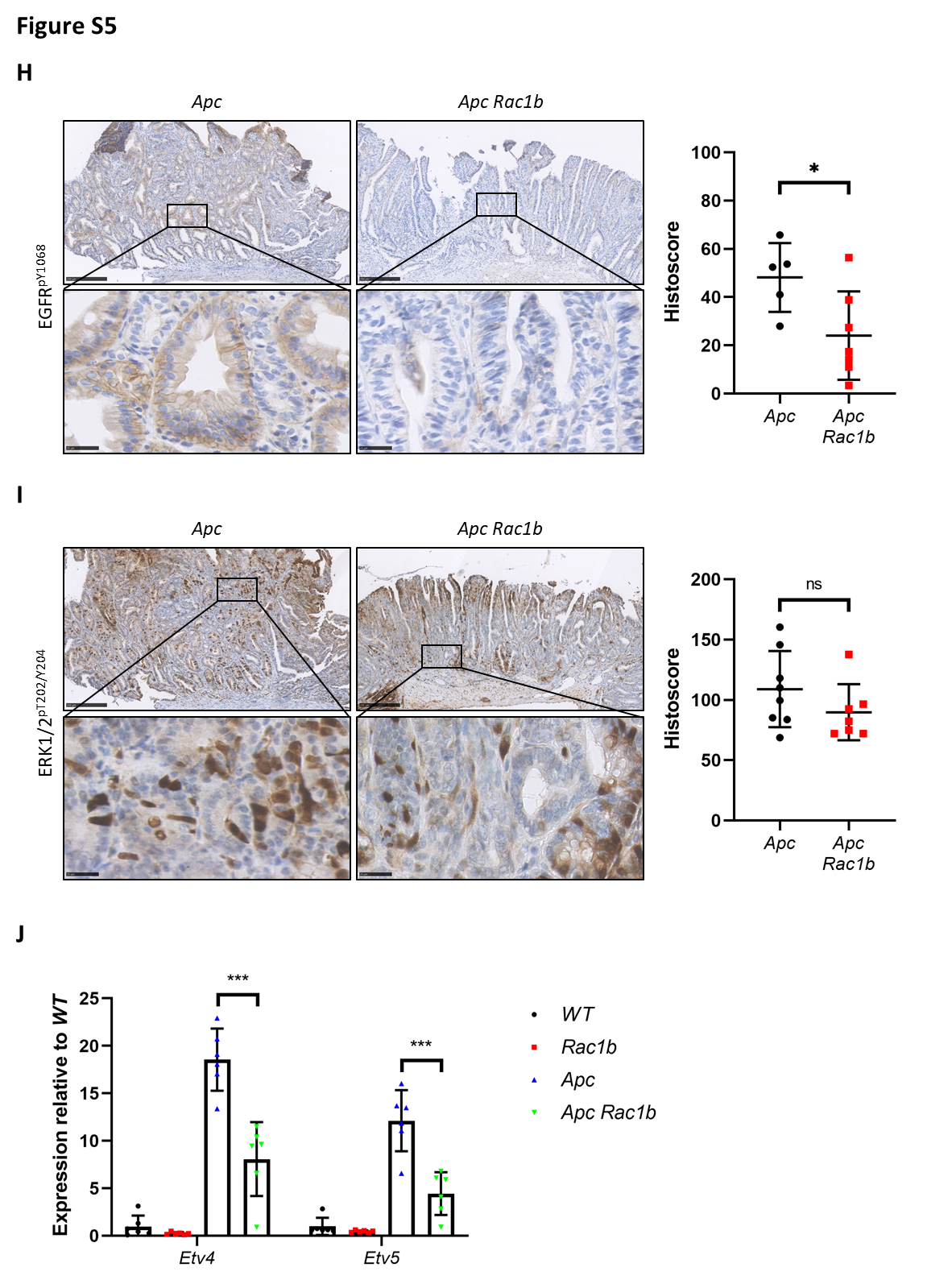
**

**Figure S5. RAC1B interacts with EGFR.** (A) Bar plots showing the top 10 most significantly enriched pathways and cellular components identified in the RAC1/RAC1B interactome. (B) Western blot analysis of RAC1B BioID experiment demonstrating EGFR pulldown. Data are representative of 3 independent technical replicate experiments. (C) qRT-PCR analysis of *Rac1b* and *Rac1* expression in CMT93 cells following 48h treatment with control and *Rac1b* targeting PMO (data are presented as mean values +/- SD; **P* = 0.0381; two way anova with Tukey multiple correction; n = 3 v 3 independent treatment experiments). (D - E) Western blot analysis of ERK1/2 and AKT activation following EGF stimulation. Vinculin or β-actin sample control blots shown. Data are representative of 3 independent technical replicate experiments. (F) CMT93 cells treated with NT or *Rac1b* PMO, serum starved and stimulated with EGF for 10 minutes before analysis by western blotting with indicated antibodies. (G) Band densitometry quantification of 4 replicate experiments (data are presented as mean values +/- SD; ***P* = 0.0024 (EGFR^pY1086^), ****P* = 0.0008 (ERK1/2^pT202/Y204^), ***P* = 0.0019 (AKT^pS473^); two tailed t test; n = 4 v 4 replicate treatment experiments). (H) IHC analysis of EGFR^pY1086^ in tumours from *Apc* and *Apc Rac1b* mice. Histoscore quantification of staining intensity (data are presented as mean values +/- SD; **P* = 0.0345; two tailed t-test; n = 5 v 7 tumours from biologically independent mice). (I) IHC analysis of ERK1/2^pT202/Y204^ in tumours from *Apc* and *Apc Rac1b* mice. Histoscore quantification of staining intensity (data are presented as mean values +/- SD; n = 8 v 7 tumours from biologically independent mice). (J) qRT-PCR analysis of *Etv4* and *Etv5* from normal tissue and tumours from *Apc* and *Apc Rac1b* mice (data are presented as mean values +/- SD; ****P* = 0.0005 (*Etv4*), ****P* = 0.0007 (*Etv5*); two tailed t test; n = matched normal SI and SI tumours from 6 v 6 biologically independent mice). Source data are provided as a Source Data file.


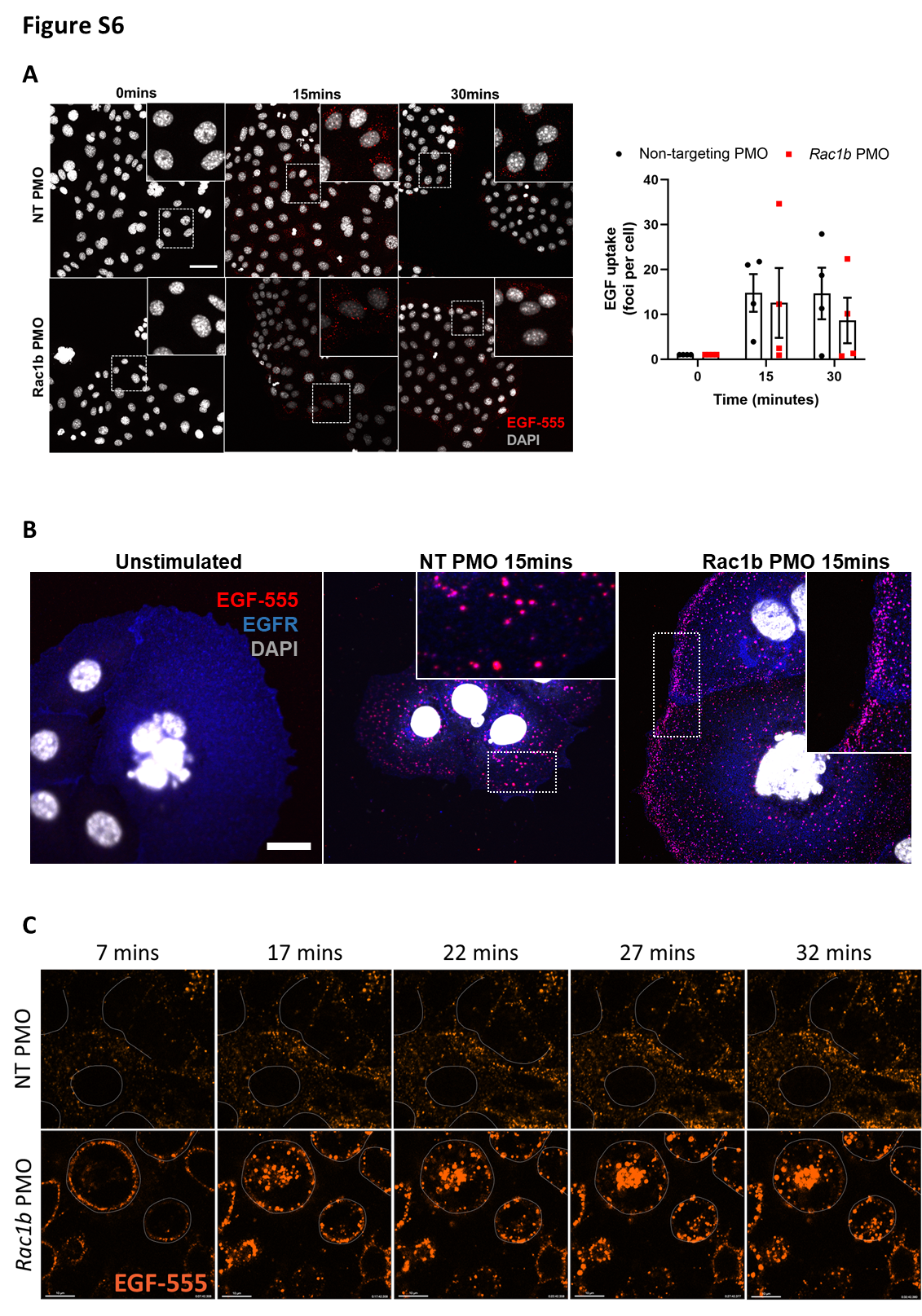

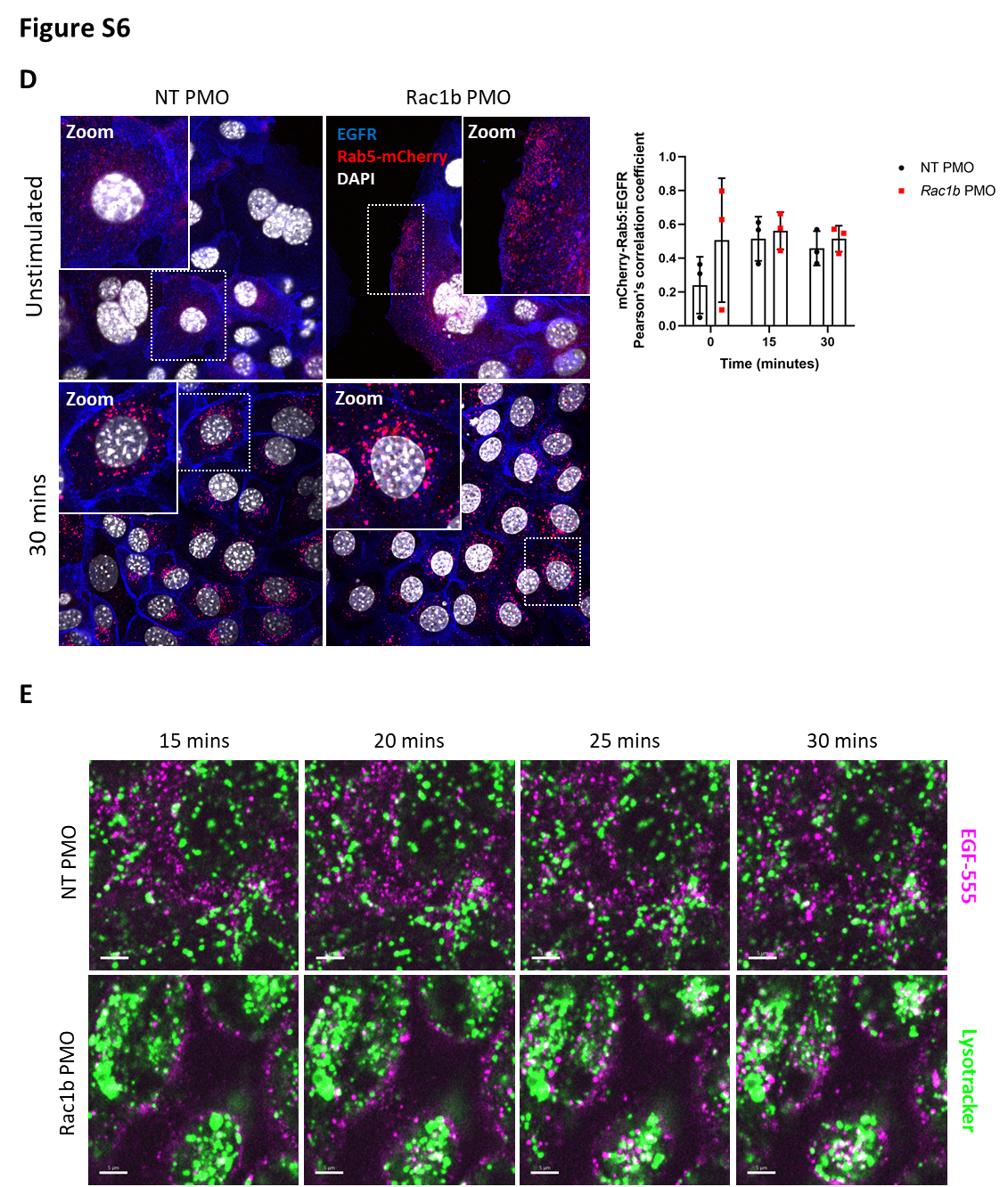

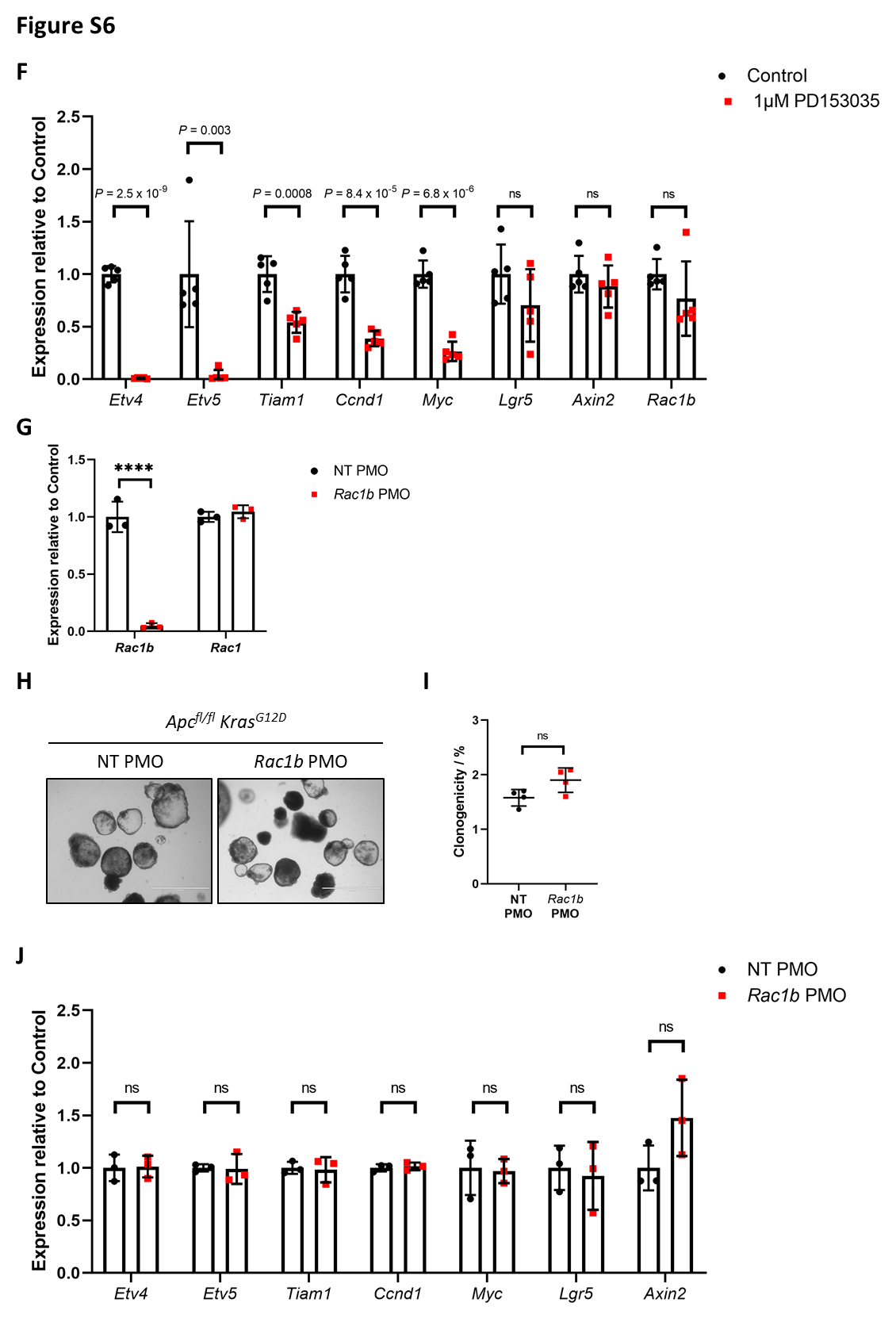

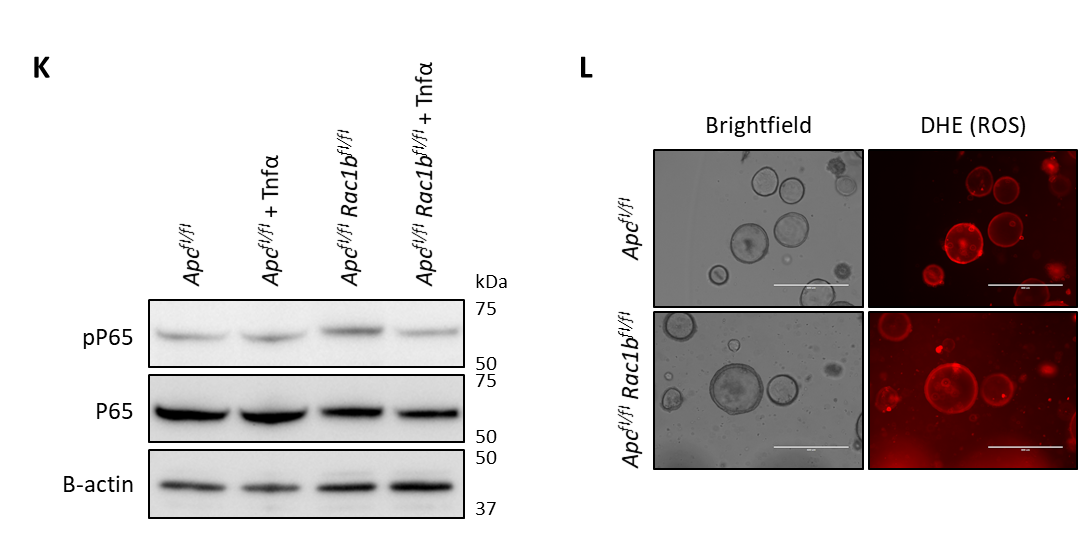


**Figure S6. RAC1B mediates EGFR signalling.** (A) Representative images of Alexa 555-EGF (red) treated control and *Rac1b* depleted CMT93 cells at indicated timepoints (left panel). Quantification of 555-EGF internalisation at indicated timepoints (data are presented as mean values +/- SEM; n = 4 v 4 technical replicate experiments) (right panel). Scale bars are 50μm. (B) Representative images of Alexa 555-EGF (pink) treated control and *Rac1b* depleted CMT93 cells stained for EGFR (blue) at indicated timepoint. (C) Live cell imaging of Alexa 555-EGF (red) treated control and *Rac1b* depleted CMT93 cells at indicated timepoints. Scale bars are 10μm. Data are representative of 3 independent technical replicate experiments. (D) Representative images of EGFR (blue) and Rab5-mCherry (pink) colocalisation in control and *Rac1b* depleted CMT93 cells at indicated timepoints (left panels). Quantification of EGFR and Rab5-mCherry colocalisation at indicated timepoints (right panel) (data are presented as mean values +/- SD; n = 3 replicate experiments). (E) High magnification images of EGF-555 (magenta) and LysoTracker green (green) live cell imaging. Co-localisation is observed as white dots. Scale bars are 5μm. Data are representative of 3 independent technical replicate experiments. (F) qRT-PCR analysis of EGFR and Wnt target genes from control and EGFR inhibitor treated *Apc^fl/fl^* organoids (data are presented as mean values +/- SD; two tailed t test; n = 5 v 5 replicate experiments). (G) qRT-PCR analysis of *Rac1b* and *Rac1* expression in control and *Rac1b* PMO treated *Apc^fl/fl^ Kras^G12D^* organoids (data are presented as mean values +/- SD; *****P* = 1.69 x 10^-6^; two way anova with Tukey multiple correction; n = 3 v 3 independent treatment experiments). (H and I) Representative images and quantification of clonogenicity experiment from control and *Rac1b* PMO treated *Apc^fl/fl^ Kras^G12D^* organoids (data are presented as mean values +/- SD; P=not significant; two tailed t test; n = 4 v 4 replicate experiments). Scale bars are 1000µm. (J) qRT-PCR analysis of EGFR and Wnt target genes from control and *Rac1b* PMO treated *Apc^fl/fl^ Kras^G12D^* organoids (data are presented as mean values +/- SD; P=not significant; two tailed t test; n = 3 v 3 biological replicate experiments). (K) P65 and pP65 Western blot analysis of *Apc^fl/fl^* and *Apc^fl/fl^ Rac1b^fl/fl^* organoids. Data are representative of 3 independent technical replicate experiments. (L) Representative images of dihydroethidium (DHE) treated *Apc^fl/fl^* and *Apc^fl/fl^ Rac1b^fl/fl^* organoids. Data are representative of 3 independent technical replicate experiments. Source data are provided as a Source Data file.

**
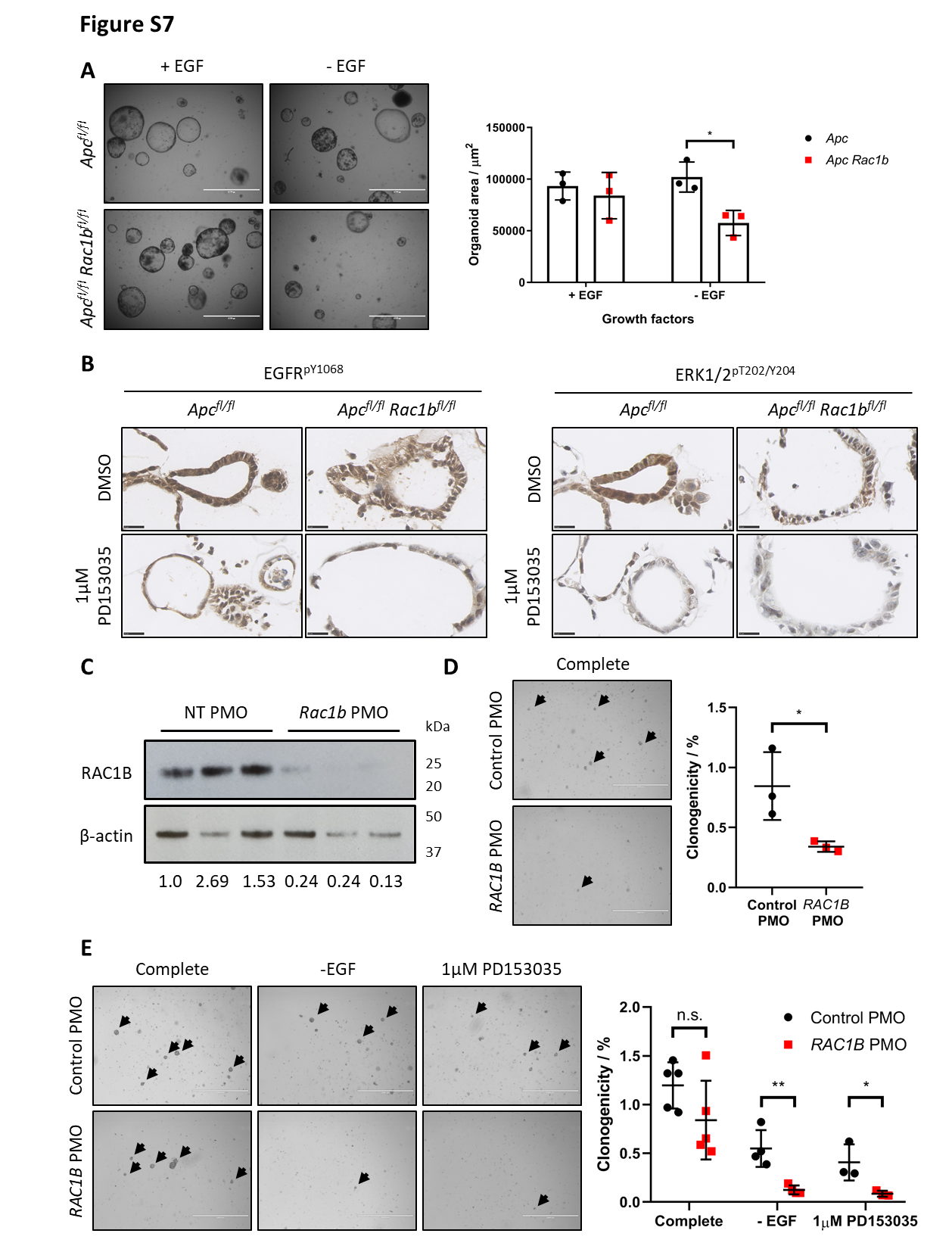

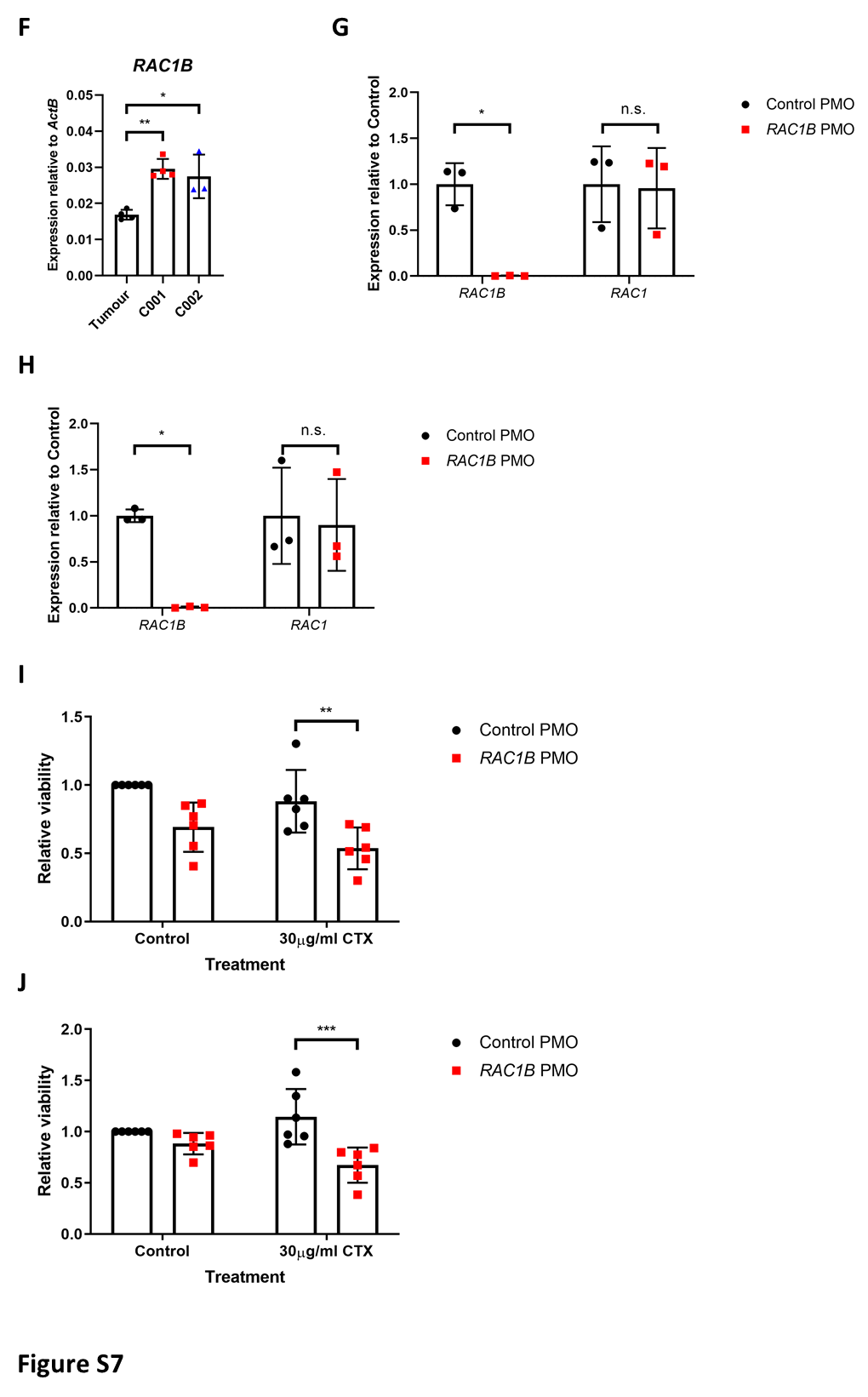
**

**Figure S7. RAC1B inhibition sensitises tumour organoids to EGFR inhibitor treatment.**

(A) Representative images of *Apc^fl/fl^* and *Apc^fl/fl^ Rac1b^fl/fl^* organoids grown with or without EGF (left panel). Scale bars are 1000µm. Quantification of organoid size 4 days after plating data are presented as mean values +/- SD; **P* = 0.0397; two way anova with Tukey multiple correction; n = organoid cultures from 3v3 mice). (B) IHC analysis of EGFR^pY1068^ and ERK1/2^pT202/Y204^ in *Apc^fl/fl^* and *Apc^fl/fl^ Rac1b^fl/fl^* organoids treated with or without 1μM PD153035 for 6 hours. Scale bars are 25µm. Data are representative of 3 independent technical replicate experiments. (C) Western blot analysis of RAC1B expression in PDOs treated with control or *RAC1B* PMO. Numbers below blots indicate densitometry measurements of RAC1B relative to β-actin normalised to lane 1 (P=0.037; two tailed t test; n=3v3 independent treatment experiments). (D) Representative images (left panel, clones indicated by black arrows) and quantification (right panel) of clonogenicity assays carried out on organoids from a benign human colonic polyp treated with control or *RAC1B* PMO (data are presented as mean values +/- SD; **P* = 0.038; two tailed t test; n=3v3 independent treatment experiments). Scale bars are 1000µm. (E) Representative images (left panel, clones indicated by black arrows) and quantification (right panel) of clonogenicity assays carried out on organoids from an invasive human colonic tumour. Organoids were treated with control or *RAC1B* PMO (data are presented as mean values +/- SD; n=5v5 independent treatment experiments). Organoids were treated with control or *RAC1B* PMO in the presence or absence of EGF ligand (data are presented as mean values +/- SD; ** *P =* 0.0047; two tailed t test; n=4v4 independent treatment experiments). Organoids were treated with control or *RAC1B* PMO in the presence or absence of the EGFR inhibitor PD153035 (data are presented as mean values +/- SD; **P* = 0.0411; two tailed t test; n=3v3 independent treatment experiments). Scale bars are 1000µm. (F) qRT-PCR analysis of *RAC1B* from an invasive human tumour organoid and 2 liver metastasis organoids (C001 and C002) (data are presented as mean values +/- SD; **P* = 0.0112, ***P* = 0.0025; one way anova with Tukey multiple correction; n = 4 vs 4 vs 3 replicate experiments). (G) qRT-PCR analysis of *RAC1B* and *RAC1* expression in control and *RAC1B* PMO treated C001 organoids (data are presented as mean values +/- SD; **P* = 0.0220; two way anova with Tukey multiple correction; n = 3 v 3 independent treatment experiments). (H) qRT-PCR analysis of *RAC1B* and *RAC1* expression in control and *RAC1B* PMO treated C002 organoids (data are presented as mean values +/- SD; **P* = 0.0404; two way anova with Tukey multiple correction; n = 3 v 3 independent treatment experiments). (I) Resazurin assay measurements of C001 organoid PDOs treated with control or *RAC1B* PMO in the presence or absence of cetuximab (data are presented as mean values +/- SD; ***P* = 0.0085; two way anova with Tukey multiple correction; n=6v6 independent treatment experiments). (J) Resazurin assay measurements of C002 organoid PDOs treated with control or *RAC1B* PMO in the presence or absence of cetuximab (data are presented as mean values +/- SD; ****P* = 0.0005; two way anova with Tukey multiple correction; n=6v6 independent treatment experiments). Source data are provided as a Source Data file.


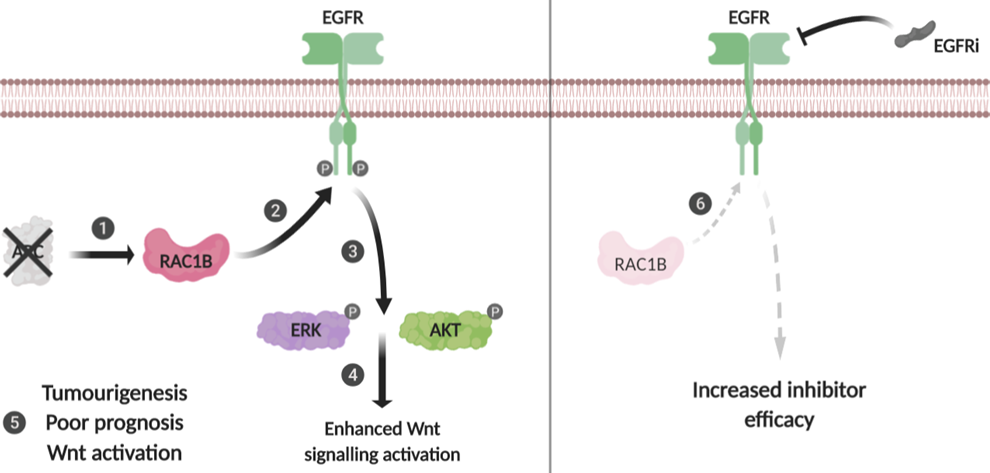


**Figure S8.** Schematic outlining function of Rac1b in promoting WNT driven tumourigenesis: (1) Following loss of *APC*, *RAC1B* expression increases. (2) RAC1B modulate EGFR phosphorylation. (3) Phosphorylation of EGFR leads to activation of MAPK signalling. (4) Via activation of EGFR signalling, RAC1B promote efficient WNT signalling activation. (5) Via these pathways RAC1B mediates tumourigenesis. (6) RAC1B depleted cells show increased sensitivity to EGFR inhibitor treatment.
