# Supplementary material for: RAC1B modulates intestinal tumourigenesis via modulation of WNT and EGFR signalling pathways
Source: Nat Commun. 2021 Apr 20;12:2335. doi: 10.1038/s41467-021-22531-3 (PMC8058071; doi:10.1038/s41467-021-22531-3)
Supplement: Supplementary file 3 — Description of Additional Supplementary Files [file 41467_2021_22531_MOESM3_ESM.docx]

Description of Additional Supplementary Files

Title: Supplementary Data 1.

Description: RNAseq analysis of Apc vs Apc Rac1b tumours. Expression values for all annotated genes are included.

Title: Supplementary Data 2.

Description: Mass spectrometry data of BioID hits from Rac1 and Rac1b BioID experiments.
