# Supplementary material for: RAC1B modulates intestinal tumourigenesis via modulation of WNT and EGFR signalling pathways
Source: Nat Commun. 2021 Apr 20;12:2335. doi: 10.1038/s41467-021-22531-3 (PMC8058071; doi:10.1038/s41467-021-22531-3)
Supplement: Supplementary file 6 — Reporting Summary [file 41467_2021_22531_MOESM6_ESM.pdf]

nature research

Corresponding author(s): Kevin Myant

Last updated by author(s): Feb 25, 2021

Reporting Summary

Nature Research wishes to improve the reproducibility of the work that we publish. This form provides structure for consistency and transparency in reporting. For further information on Nature Research policies, see our Editorial Policies and the Editorial Policy Checklist.

Statistics

For all statistical analyses, confirm that the following items are present in the figure legend, table legend, main text, or Methods section.

n/a	Confirmed	
☐	☒	The exact sample size (*n*) for each experimental group/condition, given as a discrete number and unit of measurement
☐	☒	A statement on whether measurements were taken from distinct samples or whether the same sample was measured repeatedly
☐	☒	The statistical test(s) used AND whether they are one- or two-sided<br>*Only common tests should be described solely by name; describe more complex techniques in the Methods section.*
☒	☐	A description of all covariates tested
☐	☒	A description of any assumptions or corrections, such as tests of normality and adjustment for multiple comparisons
☐	☒	A full description of the statistical parameters including central tendency (e.g. means) or other basic estimates (e.g. regression coefficient) AND variation (e.g. standard deviation) or associated estimates of uncertainty (e.g. confidence intervals)
☐	☒	For null hypothesis testing, the test statistic (e.g. *F*, *t*, *r*) with confidence intervals, effect sizes, degrees of freedom and *P* value noted<br>*Give P values as exact values whenever suitable.*
☒	☐	For Bayesian analysis, information on the choice of priors and Markov chain Monte Carlo settings
☒	☐	For hierarchical and complex designs, identification of the appropriate level for tests and full reporting of outcomes
☐	☒	Estimates of effect sizes (e.g. Cohen's *d*, Pearson's *r*), indicating how they were calculated

Our web collection on statistics for biologists contains articles on many of the points above.

Software and code

Policy information about availability of computer code

Data collection	RNAseq - Illumina HiSeq 4000, qPCR - BioRad CFX Connect, Histology - Nanozoomer.
Data analysis	Microsoft Office Excel 365, GraphPad Prism v7.0 and v8.3.1, RNAseq analysis - TrimGalore 0.6.5, cutadapt 3.2, tophat 2.1.1, cuffdiff 2.2.1, ImageJ 1.52p, kallisto 0.45, DESeq2 v1.30.1.

For manuscripts utilizing custom algorithms or software that are central to the research but not yet described in published literature, software must be made available to editors and reviewers. We strongly encourage code deposition in a community repository (e.g. GitHub). See the Nature Research guidelines for submitting code & software for further information.

Data

Policy information about availability of data

All manuscripts must include a data availability statement. This statement should provide the following information, where applicable:
- Accession codes, unique identifiers, or web links for publicly available datasets
- A list of figures that have associated raw data
- A description of any restrictions on data availability

Raw RNA sequencing data has been deposited in the Gene Expression Omnibus (GEO) with accession code GSE167876. All analyzed expression and proteomic data are available as Supplementary tables S1 and S2. Figures 1, 2, 3, 4, 5, 6, 7, S1, S2, S3, S5, S6 and S7 have associated raw data. There are no restrictions on data availability.

Field-specific reporting

Please select the one below that is the best fit for your research. If you are not sure, read the appropriate sections before making your selection.

- [x] Life sciences
- [ ] Behavioural & social sciences
- [ ] Ecological, evolutionary & environmental sciences

For a reference copy of the document with all sections, see nature.com/documents/nr-reporting-summary-flat.pdf

Life sciences study design

All studies must disclose on these points even when the disclosure is negative.

Sample size	Sample sizes for each experiment are outlined in the figure legends.<br><br>For all animal experiments, n > 3 mice were used for each experimental cohort. Power analyses were carried out prior to experiments being carried out to determine the minimum number of animals required for each experiment. These analyses were informed by previous and / or preliminary experiments.<br><br>For organoid experiments, all are derived from n = 3 or n > 3 independent experiments unless otherwise stated. Sample sizes were not statistically predetermined and were based on the results of previous experiments with these models.<br><br>For RNAseq experiments, all are derived from n = 4 independent biological samples. Sample sizes were not statistically predetermined and were based on the results of previous experiments with these models (Myant et al., 2013 Cell Stem Cell, DOI: 10.1016/j.stem.2013.04.006).
Data exclusions	No data were excluded from analysis.
Replication	All experiments (mouse, RNAseq, QRT-PCR, Western blot, organoid etc) were replicated at least 3 times using the same experimental approach or using multiple biologically independent replicates. All replication attempts were successful.
Randomization	Mice of ages 6-12 weeks of the appropriate genotype were randomly selected, with no sex-bias, for tumourigenesis studies. All mice received the same treatment (tamoxifen induction). For organoid and cell culture treatment experiments, random wells were allocated into groups prior to treatment.
Blinding	Investigators were blinded to the genotype of mice when monitoring for clinical signs. Investigators were blinded to the genotype of mice when carrying out histological analysis. IHC analysis of tumour histology was carried out using QuPath software with the investigator blinded to tumour genotype. Investigators were blinded to the treatment given to organoids and cell cultures when scoring and analysing data.

Reporting for specific materials, systems and methods

We require information from authors about some types of materials, experimental systems and methods used in many studies. Here, indicate whether each material, system or method listed is relevant to your study. If you are not sure if a list item applies to your research, read the appropriate section before selecting a response.

Materials & experimental systems

n/a	Involved in the study	
[ ]	[x]	Antibodies
[ ]	[x]	Eukaryotic cell lines
[x]	[ ]	Palaeontology and archaeology
[ ]	[x]	Animals and other organisms
[ ]	[x]	Human research participants
[x]	[ ]	Clinical data
[x]	[ ]	Dual use research of concern

Methods

n/a	Involved in the study	
[x]	[ ]	ChIP-seq
[x]	[ ]	Flow cytometry
[x]	[ ]	MRI-based neuroimaging

Antibodies

Antibodies used	BrdU, 1:500 (Bioss, bs-0489H),<br>β-catenin, 1:50 (BD Biosciences, 610154),<br>cleaved Caspase 3, 1:800 (R&D, AF835)<br>Lyz1, 1:1000 (DAKO, A009)<br>Muc2, 1:750 (Genetex, GTX100664)<br>EGFRpY1068, 1:25 (Cell Signalling, 3777S)<br>EGFRpY1068, 1:400 (Abcam, ab40815)<br>ERK1/2pT202/Y204, 1:100 (Cell Signalling, 4370S)<br>AKT, 1:2000 (Cell Signalling, 9272S)

	AKTp5473, 1:3000 (Cell Signalling, 4060S)<br>EGFR, 1:1000 (Cell Signalling, 2232S)<br>ERK1/2, 1:2000 (Cell Signalling, 4695S)<br>NF-kB p65, 1:1000 (Abcam, ab7970)<br>NF-kB p65pS536, 1:1000 (Cell Signalling, 3033S)<br>Rac1b, 1:1000 (Millipore, 09-271)<br>Vinculin 1:5000 (Abcam, ab73412)<br>Myc-tag, 1:10,000 (Cell Signalling, 2276S)<br>β-actin, 1:5000 (Cell Signalling, 4970S)<br>anti-Rabbit HRP, 1:10000 (Cell Signalling, 7074S)<br>anti-Mouse HRP, 1:10000 (Cell Signalling, 7076S)
Validation	BrdU, 1:500 (Bioss, bs-0489H): Validated by IHC staining of intestinal tissue derived from mice not injected with BrdU. There was an absence of signal in intestinal epithelial tissue in these mice. Also validated in numerous publications including DOI: 10.1016/j.stem.2013.04.006.<br>β-catenin, 1:50 (BD Biosciences, 610154). Validated on manufacturers website and in numerous publications including DOI: 10.1016/j.stem.2013.04.006.<br>cleaved Caspase 3, 1:800 (R&D): Active Caspase-3 (R&D systems, AF835): Validated on manufacturers website by immunohistochemistry analysis of human colon cancer tissue.<br>Lyz1 (DAKO, A009): Validated in various models for expected IHC expression pattern. Also validated in numerous publications including DOI:10.1038/s41467-020-20636-9.<br>Muc2 (Genetex, GTX100664): Validated in various models for expected IHC expression pattern and on manufacturers website.<br>EGFRpY1068, 1:25 (Cell Signalling, 3777S): Validated on manufacturers website by Western blot analysis of cell extracts from EGF treated cell lines. Predicted to work in human, mouse and rat samples due to sequence homology.<br>EGFRpY1068, 1:400 (Abcam, ab40815): Validated on manufacturers website by Western blot analysis of cell extracts from EGF treated cell lines. Predicted to work in mouse samples due to sequence homology.<br>ERK1/2pT202/Y204, 1:100 (Cell Signalling, 4370S): Validated on manufacturers website by Western blot analysis of cell extracts from TPA treated cell lines. Predicted to work in human, mouse and rat samples due to sequence homology.<br>AKT, 1:2000 (Cell Signalling, 9272S): Validated on manufacturers website by Western blot analysis of cell extracts from various cell lines. Predicted to work in human, mouse and rat samples due to sequence homology.<br>AKTp5473, 1:3000 (Cell Signalling, 4060S): Validated on manufacturers website by Western blot analysis of cell extracts from PDGF treated cell lines. Predicted to work in human, mouse and rat samples due to sequence homology.<br>EGFR, 1:1000 (Cell Signalling, 2232S): Validated on manufacturers website by Western blot analysis of cell extracts from various cell lines. Predicted to work in human, mouse and rat samples due to sequence homology.<br>ERK1/2, 1:2000 (Cell Signalling, 4695S): Validated on manufacturers website by Western blot analysis of cell extracts from various cell lines. Predicted to work in human, mouse and rat samples due to sequence homology.<br>NF-kB p65, 1:1000 (Abcam, ab7970): Validated on manufacturers website by Western blot analysis of cell extracts from various cell lines. Predicted to work in human, mouse and rat samples due to sequence homology.<br>NF-kB p65pS536, 1:1000 (Cell Signalling, 3033S): Validated on manufacturers website by Western blot analysis of cell extracts from TNFa treated cell lines. Predicted to work in human, mouse and rat samples due to sequence homology.<br>Rac1b (Millipore, 1:1,000): Validated on manufacturers website by Western blot analysis of cell extracts from various cell lines and in this study in Rac1b depleted organoids.<br>Vinculin (Abcam, 1:5,000): Validated on manufacturers website by Western blot analysis of cell extracts from various cell lines. Predicted to work in human, mouse and rat samples due to sequence homology.<br>Myc-tag, 1:10,000 (Cell Signalling, 2276S): Myc-Tag (Cell Signalling Technology, 2276): Validated by absence of signal in protein lysate derived from cells not transfected with Myc-tagged protein. Also validated on manufacturers website.<br>β-actin, 1:5000 (Cell Signalling, 4970S): Validated on manufacturers website by Western blot analysis of cell extracts from various cell lines. Predicted to work in human, mouse and rat samples due to sequence homology.

Eukaryotic cell lines

Policy information about cell lines

Cell line source(s)	All organoid lines were derived from mice / CRC patients during the course of this study. CMT93 cell lines were kindly provided by Dr Susan Farrington (IGMM, Edinburgh) and were originally obtained from ATCC (CMT-93 (ATCC® CCL-223™)).
Authentication	Cells were not authenticated independently.
Mycoplasma contamination	Cell lines and organoid cultures were routinely tested for Mycoplasma contamination and found to be negative.
Commonly misidentified lines<br>(See ICLAC register)	Not used.

Animals and other organisms

Policy information about studies involving animals; ARRIVE guidelines recommended for reporting animal research

Laboratory animals	Mice were bred at the animal facilities of the University of Edinburgh. Colonies had a mixed background (50% C57Bl6J, 50% S129). The genetic alleles used for this study were as follows: villinCreER, Apc (floxed), ASF/SF2 (Srsf1 floxed), Kras (G12D), IKK2ca, p53 (floxed). Both genders of mice were used for all experiments at an age of between 6 and 12 weeks once they had reached a minimum weight of 20 g. At experiment endpoints, mice were humanely sacrificed by cervical dislocation (CD) in line with UK Home Office regulations. Mice were maintained in a temperature (20-26C) and humidity (30%-70%) controlled environment.
Wild animals	Not used in this study.
Field-collected samples	Not used in this study.
Ethics oversight	All animal experiments were performed in accordance with a UK Home Office licence (Project License 70/8885), and were subject to review by the animal welfare and ethics board of the University of Edinburgh

Note that full information on the approval of the study protocol must also be provided in the manuscript.

Human research participants

Policy information about studies involving human research participants

Population characteristics	This study did not involve human research participants but utilised human derived biospecimens to generate primary organoids. Researchers were blinded to the identification of donors.
Recruitment	Patients were recruited as having primary colorectal tumours. No biases were present that might likely impact results.
Ethics oversight	Ethical approval for human CRC organoid derivation was carried out under NHS Lothian Ethical Approval Scottish Colorectal Cancer Genetic Susceptibility Study 3 (SOCCS3) (REC reference: 11/SS/0109).

Note that full information on the approval of the study protocol must also be provided in the manuscript.
